# Three New Malyngamides from the Marine Cyanobacterium *Moorea producens*

**DOI:** 10.3390/md15120367

**Published:** 2017-11-29

**Authors:** Kosuke Sueyoshi, Aki Yamano, Kaori Ozaki, Shimpei Sumimoto, Arihiro Iwasaki, Kiyotake Suenaga, Toshiaki Teruya

**Affiliations:** 1 Faculty of Education, University of the Ryukyus, 1 Senbaru, Nishihara, Okinawa 903-0213, Japan; kosuke.sueyshi@gmail.com (K.S.); yamano@jim.u-ryukyu.ac.jp (A.Y.); k168239@eve.u-ryukyu.ac.jp (K.O.); 2 Department of Chemistry, Keio University, 3-14-1 Hiyoshi, Kohoku-ku, Yokohama, Kanagawa 223-8522, Japan; e083420@yahoo.co.jp (S.S.); a.iwasaki@chem.keio.ac.jp (A.I.); suenaga@chem.keio.ac.jp (K.S.)

**Keywords:** marine cyanobacteria, malyngamides, glucose uptake, AMPK

## Abstract

Three new compounds of the malyngamide series, 6,8-di-*O*-acetylmalyngamide 2 (**1**), 6-*O*-acetylmalyngamide 2 (**2**), and *N*-demethyl-isomalyngamide I (**3**), were isolated from the marine cyanobacterium *Moorea producens*. Their structures were determined by spectroscopic analysis and chemical derivatization and degradation. These compounds stimulated glucose uptake in cultured L6 myotubes. In particular, 6,8-di-*O*-acetylmalyngamide 2 (**1**) showed potent activity and activated adenosine monophosphate-activated protein kinase (AMPK).

## 1. Introduction

**Figure 1:**
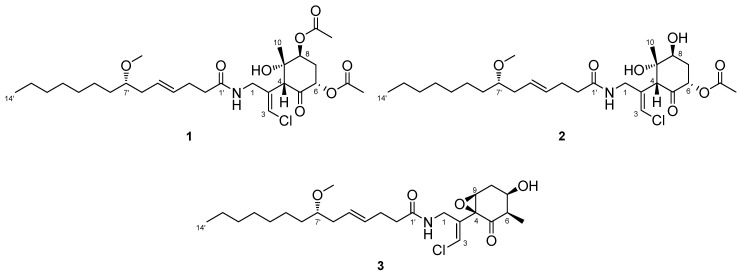
Structures of new malyngamides **1–3**.

The ocean covers more than 70% of the Earth’s surface and hosts huge biological and chemical diversity. Because marine environmental conditions are quite different from terrestrial ones, natural products from marine organisms have unique structures and biological activities. Marine cyanobacteria, in particular, are known to produce various secondary metabolites and have been recognized as a source of pharmaceutical lead compounds [1,2,3]. For example, bisebromoamide, isolated from *Lyngbya* sp., showed potent cytotoxicity against HeLa S_3_ cells [4]. Bisebromoamide inhibited the phosphorylation of extracellular signal-regulated protein kinase (ERK) and was identified as an actin filament stabilizer [5]. Meanwhile, hoshinolactam was found to possess both a cyclopropane ring and γ-lactam ring, and exhibited potent antitrypanosomal activity without cytotoxicity against human fetal lung fibroblast MRC-5 cells [6]. The malyngamide series of natural products have been isolated from various marine filamentous cyanobacteria. Malynagmide A, the first compound of this group, was isolated from *Lyngbya majuscule* in 1979 [7]; since then, over 30 malyngamide analogs have been isolated [8]. As part of our ongoing effort to identify novel bioactive natural products, we have focused on the constituents of marine cyanobacteria and isolated odoamide [9,10] and odobromoamide [11]. We recently discovered three new malyngamides, 6,8-di-*O*-acetylmalyngamide 2 (**1**), 6-*O*-acetylmalyngamide 2 (**2**), and *N*-demethyl-isomalyngamide I (**3**), from the Okinawan cyanobacterium belonging to the genus *Moorea producens*. (Figure 1). Herein, we report the isolation, structure determination, and biological evaluation of these compounds.

## 2. Results

### 2.1. 6,8-Di-O-Acetylmalyngamide 2 *(**1**)* and 6-O-Acetylmalyngamide 2 *(**2**)*

The marine cyanobacterium *M. producens* (30 g, wet weight) was collected at Bise, Okinawa Prefecture, Japan, and extracted with methanol. The extract was filtered and concentrated, and then the residue was partitioned between EtOAc and H_2_O. The organic layer was further partitioned between 90% aqueous MeOH and *n*-hexane. The material obtained from the 90% aqueous portion was fractionated by octadecylsilyl (ODS) column chromatography and subjected to reversed-phase high-performance liquid chromatography (HPLC) to give compound **1** (12.0 mg), **2** (26.8 mg), and **3** (12.6 mg).

**Figure 2:**
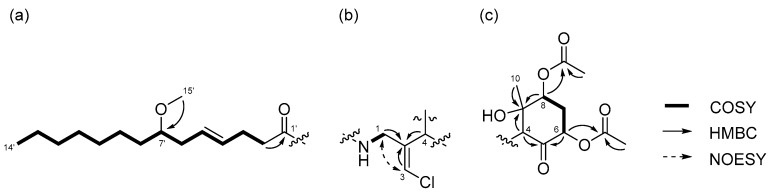
Partial structures of compound **1**: (**a**) lyngbic acid moiety; (**b**) chloromethylene moity; (**c**) cyclohexanone ring moiety of **1**. Wavy lines were used to indicate partial structures of **1**.

Compound **1** was obtained as a colorless oil. The molecular formula of **1** was determined to be C_29_H_46_ClNO_8_ on the basis of the ^13^C NMR spectrum (29 carbon signals, Figure S2) and high-resolution electrospray ionization mass spectrometry (HRESIMS) (*m*/*z* 572.2989 [M + H]^+^, calcd. 572.2985). The nuclear magnetic resonance (NMR) data for **1** are summarized in Table 1. These data indicated the presence of a secondary amide (δ_H_ 6.68, δ_C_ 173.8), disubstituted olefin (δ_H_ 5.45, 5.47, δ_C_ 128.3, 130.7), vinyl chloride (δ_H_ 6.29, δ_C_ 121.6) [12], and *O*-methyl group (δ_H_ 3.31, δ_C_ 56.5), which are characteristic resonances of the malyngamide series [7,13,14,15]. A detailed 2D NMR analysis using COSY, HSQC, and HMBC data (Figures S3–S5) revealed the presence of 7-methoxytetradec-4(*E*)-enoic acid (lyngbic acid, Figure 2a). The *E*-geometry of the C-4’/C-5’ olefin was assigned based on the comparison of the ^13^C NMR chemical shifts of C-3’ (δ_C_ 28.7) and C-6’ (δ_C_ 36.3) with those of other known malyngamides [7,13,14,15].

**Figure 3:**
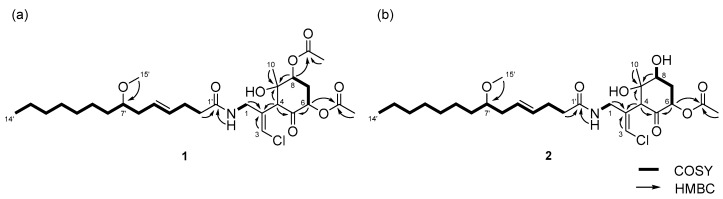
Gross structures of (**a**) compound **1** and (**b**) **2**.

The structure of the remaining C_14_H_19_ClNO_6_ unit was determined as follows. COSY correlation between H-1/NH and HMBC correlations between H-1/C-2, H-3/C-2, and H-4/C-2 led to the partial structure C-1 to C-4 containing the chloromethylene moiety (Figure 2b). The geometry of the vinyl chloride was determined to be *E* by NOESY correlation between H-3/H-1. Additionally, COSY correlations between H-6/H-7 and H-7/H-8, and HMBC correlations between H-4/C-5, H-4/C-9, H-6/C-5, and H-8/C-9, allowed the assignment of the cyclohexanone ring. The chemical shifts of H-6 (δ_H_ 5.41), C-6 (δ_C_ 72.4), 7 and HMBC correlation from H-6 to the quaternary carbon (δ_C_ 170.3) connected the acetyl group (δ_H_ 2.16, δ_C_ 20.8) to C-6 via the oxygen atom. Similarly, another acetyl group (δ_H_ 2.19, δ_C_ 21.3) was connected to C-8 via the oxygen atom. The remaining methyl group (δ_H_ 1.26, δ_C_ 23.5) and OH group (δ_H_ 5.69) were connected to C-9 by HMBC correlations between H-10/C-9 and OH/C-9 (Figure 2c). The connections of these partial structures (Figure 2a–c) were determined on the basis of HMBC data. The NH proton showed a cross peak to the C-1’ carbonyl carbon, and H-4 correlated with the C-2 olefinic carbon. Thus, the gross structure of **1** was determined to be that shown in Figure 3a.

Compound **2** was obtained as a colorless oil. The molecular formula of **2** was determined to be C_27_H_44_ClNO_7_ on the basis of the ^13^C NMR spectrum (27 carbon signals, Figure S8) and HRESIMS (*m*/*z* 552.2692 [M + Na]^+^, calcd. 552.2699). The ^1^H NMR features of **2** (Figure S7) were very similar to those of **1**, but there was only one signal from an acetyl proton. In the ^13^C NMR spectrum of **2**, there was one less carbonyl carbon compared with that of **1**, and thus, compound **2** was thought to be a deacetylated version of compound **1**. COSY correlations between H-6/H-7, H-7/H-8, and H-8/OH, and HMBC correlations between H-6/C-5 and H-8/C-9 revealed the position of the acetyl group at C-6. Therefore, the gross structure of **2** was established to be that depicted in Figure 3b.

**Figure 4:**
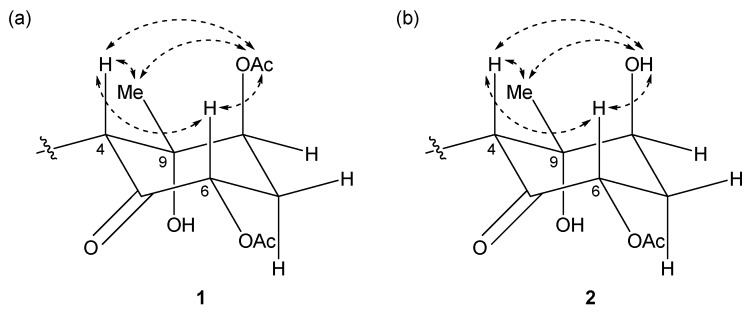
Key NOESY correlations for (**a**) compound **1** and (**b**) **2**.

The relative structures of the cyclohexanone rings in **1** and **2** were determined by NOESY experiments. The NOESY spectrum of **1** indicated that H-4, H-6, and 8-OAc were in axial positions of the ring (Figure 4a). NOESY correlations between H-4/H_3_-10 and 8-OAc/H_3_-10 indicated the methyl group at C-9 was in an equatorial position, and thus, the relative configuration of the ring moiety in **1** was 4R*, 6S*, 8S*, and 9S*. The relative configuration of the cyclohexanone ring in **2** was revealed to be 4R*, 6S*, 8S*, and 9S* by NOESY correlations (Figure 4b), and was the same as that **1**.

**Figure 5:**
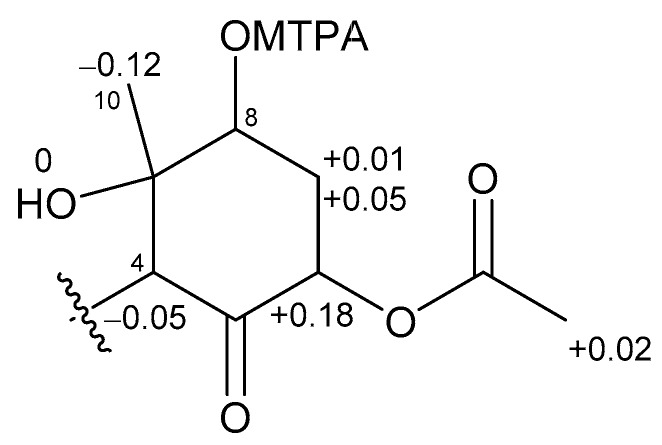
Δδ values (δ*_S_* − δ*_R_*) in ppm for the MTPA ester of **2**.

The absolute stereochemistries of the cyclohexanone rings in **1** and **2** were determined as follows. Compound **2** was treated with (*R*)- and (*S*)-MTPACl to give (*S*)- and (*R*)-MTPA esters, respectively. The ^1^H NMR chemical shifts of these esters (Figures S25 and S26) were assigned on the basis of the COSY spectrum. Calculation of the Δδ_(*S*−*R*)_ values (Figure 5) revealed that C-8 existed in the *S* configuration [16], and the absolute configuration of the ring moiety in **2** was therefore determined to be 4*R*, 6*S*, 8*S*, and 9*S*. Compound **2** was derivatized with Ac_2_O to give an acetylated derivative of compound **2**. The optical rotation value of this compound (${\left[\alpha \right]}_{D}^{25}$ +5.5) was identical to that of **1** (${\left[\alpha \right]}_{D}^{24}$ +4.8). Thus, the absolute configuration of the cyclohexanone ring in **1** was determined to be 4*R*, 6*S*, 8*S*, and 9*S*.

To confirm the absolute configuration of C-7’ in **2**, compound **2** was hydrolyzed under basic conditions to yield lyngbic acid. The optical rotation of the product (${\left[\alpha \right]}_{D}^{26}$ −10.8) was comparable to the reported value for 7(*S*)-methoxytetradec-4(*E*)-enoic acid (${\left[\alpha \right]}_{D}^{26}$ −11.1 [17]), thus establishing the *S* configuration at the C-7’ position in **2**. Because the ^1^H NMR spectrum (Figure S24) and optical rotation of the acetylated compound of **2** were identical to those of **1** described above, the absolute configuration of C-7’ in **1** was determined to be *S*. Therefore, the complete stereostructures of compound **1** and **2** were established to be those shown in Figure 1.

### 2.2. N-Demethyl-isomalyngamide I *(**3**)*

**Figure 6:**
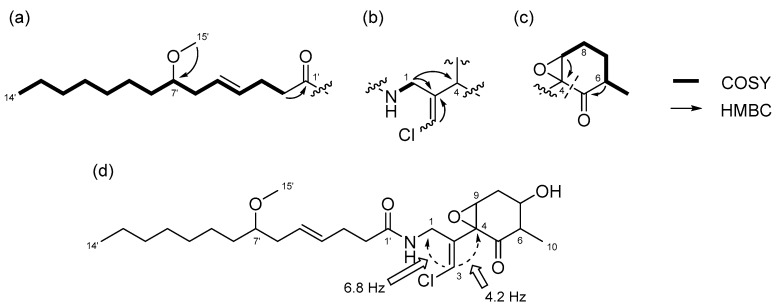
Partial structures and gross structure of compound **3**: (**a**) lyngbic acid moiety; (**b**) chloromethylene moiety; (**c**) cyclohexanone ring moiety; (**d**) gross structure of **3**.

Compound **3** was obtained as a colorless oil. The molecular formula of **3** was determined to be C_25_H_40_ClNO_5_ on the basis of the ^13^C NMR spectrum (25 carbon signals, Figure S14) and HRESIMS (*m*/*z* 460.2692 [M + H]^+^, calcd. 470.2668). The NMR data for **3** are summarized in Table 2. The 1D and 2D NMR spectra of **3** revealed that it had a lyngbic acid moiety (Figure 6a), like compound **1** and **2**. The structure of the remaining C_10_H_13_ClNO_3_ unit was determined as follows. COSY correlation between H-1/NH and HMBC correlations between H-1/C-2, H-1/C-4, and H-3/C-2 led to the partial structure C-1 to C-4 containing a chloromethylene moiety (Figure 6b). Because the geometry of the vinyl chloride could not be determined by NOESY experiments, we conducted HSQMBC NMR experiments [18]. We observed a 6.8 Hz ^3^*J* coupling from H-3 to C-1 and 4.2 Hz ^3^*J* coupling from H-3 to C-4. These coupling constants and comparison with the reported results for malyngamide R [14] revealed the *E* geometry of this double bond. Additionally, COSY correlations between H-10/H-6, H-6/H-7, H-7/H-8, and H-8/H-9, and HMBC correlations between H-6/C-5 and H-9/C-4 led to the partial structure from C-5 to C-4 (Figure 6c). The chemical shifts of H-7 (δ_H_ 3.81) and C-7 (δ_C_ 68.7) were consistent with the presence of a hydroxy group at C-7. The remaining component and the high-field chemical shifts of C-4 and C-9 (δ_C_ 61.4 and 62.1, respectively) indicated the presence of an epoxy group at C-4 and C-9 [19,20]. Although HMBC correlations between H-6/C-5 and H-9/C-5 were not observed, C-4 and C-5 should be connected considering the degree of unsaturation of **3**. Thus, it became apparent that compound **3** had a cyclohexanone ring, and the gross structure of **3** was determined to be that displayed in Figure 6d.

**Figure 7:**
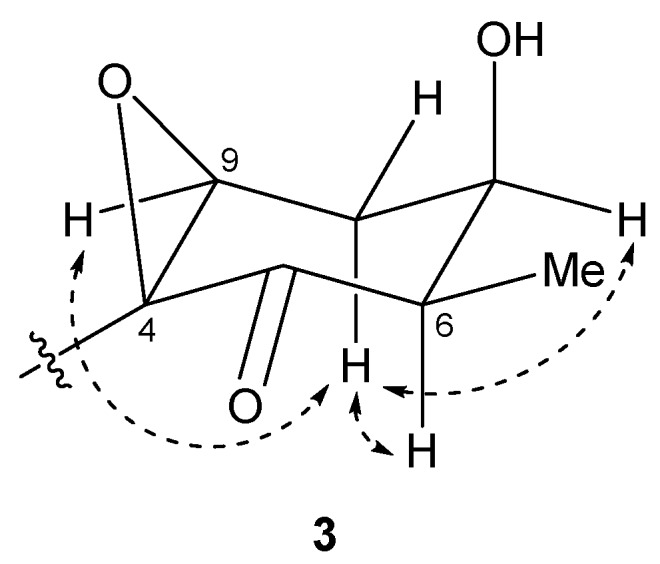
Key NOESY correlations for compound **3**.

The relative structure of the cyclohexanone ring in **3** was determined by a NOESY experiment. The NOESY spectrum of **3** (Figure S18) indicated that H-6 and H-8b were in axial positions of the ring. The coupling constant of H-9 (2.6 Hz) and NOESY correlations between H-8b/H-7 and H-8b/H-9 indicated that H-7 and H-9 were in equatorial positions of the ring. Therefore, the relative configuration of the ring moiety in **3** was deduced to be 4S*, 6R*, 7R*, and 9S* (Figure 7).

**Figure 8:**
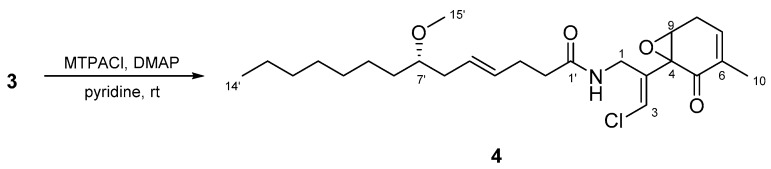
Structure of compound **4**.

To determine the absolute configuration of C-7, **3** was treated with MTPACl. However, the MTPA ester of **3** was not obtained; compound **4** was obtained instead (Figure 8). Accordingly, the absolute stereochemistry of the cyclohexanone ring in **3** was not determined. The absolute configuration of C-7’ was established to be *S* using the same method as described above for **2**.

Structurally similar compounds, malyngamide I [21] and 8-*epi*-malyngamide C [20], have been reported. The formation of the carbon framework of compound **3** is predicted to proceed in a similar fashion to those of 8-*epi*-malyngamide C and jamaicamides, and the branching methyl group C-6 should originate from methionine [12].

### 2.3. Biological Activities

**Figure 9:**
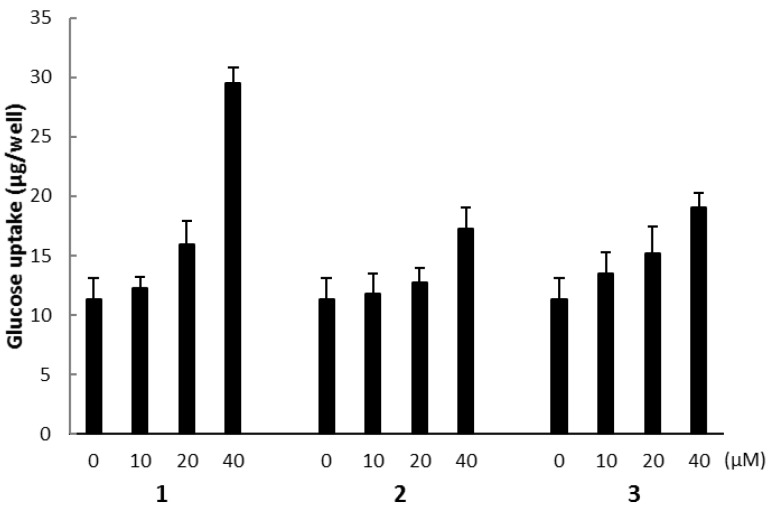
The effect of compounds **1**, **2**, and **3** on glucose uptake in cultured L6 myotubes. Cells were preincubated in Krebs-Henseleit-HEPES buffer (KHH buffer) without glucose for 2 h. They were then incubated in KHH buffer containing 5 mM glucose with the indicated concentrations of compounds for 16 h. Glucose uptake was measured using a Glucose C-II Test kit. Values are the mean ± SD of quadruplicate determinations.

**Figure 10:**
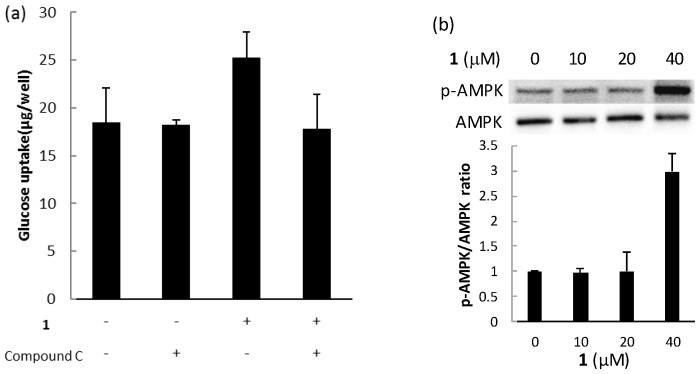
The effect of compound **1** on the AMPK pathway in cultured L6 myotubes. (**a**) Cells were preincubated in KHH buffer without glucose for 2 h. They were then incubated in KHH buffer containing 5 mM glucose with or without 40 μM of compound **1** and with or without 30 μM of compound C for 8 h. Glucose uptake was measured using a Glucose C-II Test kit. Values are the mean ± SD of quadruplicate determinations. (**b**) Cells were treated with the indicated concentrations of compound **1**. The lysates were analyzed by western blotting with anti-phosphorylated AMPK (p-AMPK) and anti-AMPK antibodies. Immunoreactive bands were quantified using Image Lab software and ratios of p-AMPK/AMPK are shown. The ratio of the control (0 μM of compound **1**) was regarded as 1. Values are the mean ± SD of quadruplicate determinations.

The biological activities of compounds **1**, **2**, and **3** were evaluated using a glucose uptake assay in cultured L6 myotubes. Compound **1** stimulated glucose uptake in a dose-dependent and insulin-independent manner, and compounds **2** and **3** showed weak activity for glucose uptake (Figure 9). To confirm the involvement of AMP-activated protein kinase (AMPK), which increases insulin-independent glucose uptake in skeletal muscle [22,23], we examined the effect of compound C, a selective AMPK inhibitor and performed western blotting with anti-AMPK and anti-phosphorylated AMPK (p-AMPK) antibodies. Compound C markedly lowered glucose uptake stimulated by compound **1** in cultured L6 myotubes (Figure 10a). The expression of p-AMPK (the activated form of AMPK) increased in the cells treated with 40 μM of **1** (Figure 10b). These results indicate that compound **1** stimulated glucose uptake in cultured L6 myotubes via the AMPK pathway, regulating cellular metabolism.

## 3. Experimental Section

### 3.1. General Experimental Procedures

Chemicals and solvents were the best grade available and used as received from commercial sources. Rat L6 myoblasts were purchased from JCRB Cell Bank (Osaka, Japan). Optical rotations were measured on a JASCO P-1010 polarimeter (JASCO Corporation, Tokyo, Japan). IR spectra were measured on a JASCO FT/IR-6100 spectrometer (JASCO Corporation, Tokyo, Japan). All NMR spectra were recorded on a Bruker AVANCE III 500 NMR spectrometer (500 and 125 MHz for ^1^H and ^13^C NMR, respectively, Bruker BioSpin Corporation, Billerica, MA, USA). Chemical shifts were reported as δ values in parts per million (ppm) relative to the residual solvent signals (CD_3_Cl: δ_H_ 7.26, δ_C_ 77.0), and coupling constants were in hertz (Hz). ESIMS data were obtained using a Waters Quattro micro API mass spectrometer (Waters Corporation, Milford, MA, USA), HRESIMS data were obtained using a Waters Micromass Q-TOF spectrometer (Waters Corporation, Milford, MA, USA). HPLC was carried out on a JASCO PU-2080 Plus Intelligent HPLC pump (JASCO Corporation, Tokyo, Japan) and a JASCO UV-2075 Plus Intelligent UV/VIS detector (JASCO Corporation, Tokyo, Japan). Absorbance of assay mixture was completed using a BioTek ELx800 absorbance microplate reader (BioTek Instruments Inc., Winooski, VT, USA).

### 3.2. Collection, Extraction, and Isolation

Samples of the marine cyanobacterium, *Moorea producens* were collected by hand from the coast of Bise, Okinawa Prefecture, Japan, in April 2016. The cyanobacterium was identified by 16S rRNA sequence analysis. Approximately 30 g (wet weight) of the samples were extracted with MeOH (1.0 L). The extract was filtered, and the filtrate was concentrated. The residue was partitioned between H_2_O (0.2 L) and EtOAc (0.2 L × 3). The material obtained from the organic layer was further partitioned between 90% aqueous MeOH (0.1 L) and *n*-hexane (0.1 L × 3). The aqueous MeOH fraction (0.23 g) was separated by column chromatography on ODS (2.0 g) using 60% aqueous MeOH, 80% aqueous MeOH, and MeOH. The fraction (131.4 mg) eluted with 80% aqueous MeOH was subjected to reversed-phase HPLC [Cosmosil 5C_18_-AR-II (20 mm × 250 mm), 85% MeOH at 5.0 mL/min, and UV detection at 215 nm] to give five fractions (Fractions 1–5). Fraction 1 was subjected to further HPLC [Cosmosil 5C_18_-AR-II (20 mm × 250 mm), 80% MeOH at 5.0 mL/min, and UV detection at 215 nm] to yield compound **2** (26.8 mg, *t*_R_ = 34.2 min). Fraction 2 was subjected to further HPLC [Cosmosil 5C_18_-AR-II (20 mm × 250 mm), 70% MeCN at 5.0 mL/min, and UV detection at 215 nm] to yield compound **3** (12.6 mg, *t*_R_ = 38.4 min) and two fractions (Fractions 2-1 and 2-2). Fraction 2-2 was further subjected to HPLC [Cosmosil π NAP (20 mm × 250 mm), 65% MeCN at 5.0 mL/min, and UV detection at 215 nm] to yield compound **1** (12.0 mg, *t*_R_ = 35.1 min).

*6,8-Di-O-Acetylmalyngamide* 2 (**1**): Colorless oil; ${\left[\alpha \right]}_{D}^{24}$ +4.8 (*c* 1.20, MeOH); IR (neat) 3302, 2929, 2856, 1731, 1651, 1539, 1445, 1373, 1232, 1132, 1096 cm^−1^; ^1^H NMR, ^13^C NMR and HMBC data, see Table 1; HRESIMS *m*/*z* 572.2989 [M + H]^+^ (calcd. for C_29_H_47_ClNO_8_ 572.2985).

*6-O-Acetylmalyngamide 2* (**2**): Colorless oil; ${\left[\alpha \right]}_{D}^{23}$ +22.9 (*c* 2.68, MeOH); IR (neat) 3375, 2929, 2856, 1730, 1650, 1540, 1446, 1375, 1239, 1066 cm^−1^; ^1^H NMR, ^13^C NMR and HMBC data, see Table 1; HRESIMS *m*/*z* 552.2692 [M + Na]^+^ (calcd. for C_27_H_44_ClNO_7_Na 552.2699).

*N-Demethyl-isomalyngamide I* (**3**): Colorless oil; ${\left[\alpha \right]}_{D}^{23}$ +108.2 (*c* 1.12, MeOH); IR (neat) 3313, 2928, 2856, 1714, 1647, 1541, 1456, 1433, 1094 cm^−1^; ^1^H NMR, ^13^C NMR and HMBC data, see Table 2; HRESIMS *m*/*z* 470.2692 [M + H]^+^ (calcd. for C_25_H_41_ClNO_5_ 470.2668).

### 3.3. Identification of the Marine Cyanobacterium

Morphological observation was performed using a phase contrast microscopy ECLIPSE Ti-S (Nicon, Tokyo, Japan). The mean cell size and standard deviation of 50 cells were measured. The cell width was observed 36.3 ± 2.0 μm and length was observed 4.0 ± 0.9 μm. The cells were surrounded by thick (2.5–14.5 µm) firm and laminated sheaths. These morphological characters were consistent with description of *Moorea producens* [24]. Therefore, the marine cyanobacterium was identified as *M. producens*.

### 3.4. Preparation of MTPA Esters of ***2***

Compound **2** (1.1 mg) was reacted with *R*-MTPACl (10 μL) and DMAP (1.1 mg) in pyridine (50 μL), and the mixture was stirred for 5 h at room temperature. The reaction mixture was concentrated, and the residue was partitioned between EtOAc and 0.1 M NaHCO_3_ (1:1). The extract obtained from the organic layer was subjected to reversed-phase HPLC [Cosmosil 5C_18_-AR-II (20 mm × 250 mm), 85% MeCN at 5.0 mL/min, and UV detection at 215 nm] to yield *S*-MTPA ester (0.8 mg). Using the same procedure as described above, *R*-MTPA (0.5 mg) ester was obtained from **2** (1.0 mg).

*S*-MTPA ester: ^1^H NMR (500 MHz, CDCl_3_) δ 4.03 (H-4), 5.29 (H-6), 2.41 (H-7a), 2.66 (H-7b), 5.28 (H-8), 1.12 (H-10), 2.15 (6-OAc); ESIMS *m*/*z* [M + Na]^+^ 768.3.

*R*-MTPA ester: ^1^H NMR (500 MHz, CDCl_3_) δ 4.08 (H-4), 5.11 (H-6), 2.40 (H-7a), 2.61 (H-7b), 5.21 (H-8), 1.24 (H-10), 2.13 (6-OAc); ESIMS *m*/*z* [M + Na]^+^ 768.3.

### 3.5. Preparation of Acetylated Compound ***2***

Compound **2** (5.0 mg) was reacted with Ac_2_O (50 μL) in pyridine (50 μL), and the mixture was stirred for 2 h at room temperature. The reaction mixture was subjected to reversed-phase HPLC [Cosmosil 5C_18_-AR-II (10 mm × 250 mm), 85% MeOH at 4.0 mL/min, and UV detection at 215 nm] to yield acetylated compound of **2** (4.7 mg).

Acetylated compound of **2**: ${\left[\alpha \right]}_{D}^{25}$ +5.5 (*c* 1.20, MeOH); ^1^H NMR (500 MHz, CDCl_3_) δ 6.63 (1H, t, *J* = 6.0 Hz), 6.29 (1H, d, *J* = 1.6 Hz), 5.69 (1H, s), 5.47 (1H, m), 5.45 (1H, m), 5.41 (1H, dd, *J* = 12.9, 6.7 Hz), 5.11 (1H, t, *J* = 2.8 Hz), 4.27 (1H, s), 4.23 (1H, dd, *J* = 16.5, 6.7 Hz), 3.95 (1H, ddd, *J* = 16.5, 5.8, 2.0 Hz), 3.31 (3H, s), 3.17 (1H, m), 2.60 (1H, dt, *J* = 13.3, 2.6 Hz), 2.32 (1H, m), 2.28 (2H, m), 2.27 (2H, m), 2.19 (3H, s), 2.18 (2H, m), 2.16 (3H, s), 1.42 (2H, m), 1.27 (10H, m), 1.26 (3H, s), 0.87 (3H, t, *J* = 6.7 Hz); ESIMS *m*/*z* [M + H]^+^ 572.3.

### 3.6. Base Hydrolysis of Compounds ***2*** and ***3***

Compound **2** (7.3 mg) was dissolved in a 5.0 mL solution of 10% KOH in 80% aqueous EtOH and refluxed for 14 h. The reaction mixture was concentrated, and the residue was partitioned between EtOAc and H_2_O. The organic layer was subjected to reversed-phase HPLC [Cosmosil 5C_18_-AR-II (10 mm × 250 mm), 80% MeOH with 0.1% TFA at 5.0 mL/min, and UV detection at 215 nm] to yield lyngbic acid (1.6 mg). Using the same procedure as described above, lyngbic acid (4.7 mg) ester was obtained from **3** (13.5 mg).

Lyngbic acid from **2**: ${\left[\alpha \right]}_{D}^{26}$ −10.8 (*c* 0.16, CHCl_3_); ^1^H NMR (500 MHz, CDCl_3_) δ 5.49 (2H, m), 3.32 (3H, s), 3.15 (1H, m), 2.42 (2H, m), 2.35 (2H, m), 2.19 (2H, m), 1.43 (2H, m), 1.27 (10H, m), 0.88 (3H, t, *J* = 6.8 Hz).

Lyngbic acid from **3**: ${\left[\alpha \right]}_{D}^{26}$ −19.6 (*c* 0.47, CHCl_3_); ^1^H NMR (500 MHz, CDCl_3_) δ 5.49 (2H, m), 3.32 (3H, s), 3.15 (1H, m), 2.42 (2H, m), 2.35 (2H, m), 2.19 (2H, m), 1.43 (2H, m), 1.27 (10H, m), 0.88 (3H, t, *J* = 6.8 Hz).

### 3.7. Preparation of MTPA Ester of Compound ***3***

Compound **3** (7.3 mg) was reacted with *R*-MTPACl (10 μL) and DMAP (0.5 mg) in pyridine (50 μL), and the mixture was stirred for 3 h at room temperature. The reaction mixture was concentrated, and the residue was partitioned between EtOAc and 0.1M NaHCO_3_ (1:1). The extract obtained from the organic layer was subjected to reversed-phase HPLC [Cosmosil 5C_18_-AR-II (20 mm × 250 mm), 85% MeOH at 5.0 mL/min, and UV detection at 215 nm] to yield compound **4** (2.3 mg).

Compound **4**: ${\left[\alpha \right]}_{D}^{23}$ +40.0 (*c* 0.23, MeOH); ^1^H NMR, ^13^C NMR and HMBC data, see Table S1; HRESIMS *m*/*z* 474.2381 [M + Na]^+^ (calcd. for C_25_H_38_ClNO_4_Na 474.2382).

### 3.8. Culture of L6 Myoblasts

L6 myoblasts (5 × 10^3^ cells/well in 96-well plates or 2 × 10^5^ cells/well in 60-mm culture dishes) were maintained in high glucose Dulbecco’s modified Eagle’s medium (DMEM) containing 10% fetal bovine serum (FBS), streptomycin (100 µg/mL), and penicillin G (100 units/mL) at 37 °C with 5% CO_2_. After reaching 80% confluence, the cells were cultured in low glucose DMEM containing 2% FBS for 1 week to differentiate into myotubes. The medium was renewed every 2 days.

### 3.9. Determination of Glucose Uptake

L6 myotubes were incubated in filter-sterilized Krebs-Henseleit buffer (1.2 mM MgSO_4_, 1.2 mM KH_2_PO_4_, 4.7 mK KCl, 119 mM NaCl, 2.5 mM CaCl_2_·2H_2_O, and 25 mM NaHCO_3_, pH 7.4) containing 0.1% bovine serum albumin (BSA), 10 mM HEPES, and 2 mM sodium pyruvate (KHH buffer) for 2 h. The myotubes were then cultured in KHH buffer containing 5 mM glucose with or without compounds **1**, **2**, and **3** (10–40 µM) for 16 h and without or with compound C (30 µM), an AMPK inhibitor for 6 h. Nepodin [25] was used as a positive control, and DMSO alone was used as a negative control. The concentrations of glucose remaining in KHH buffer were determined by a commercial assay kit (Glucose CII-Test Wako) and a microplate reader at 490 nm. The amounts of glucose uptake by myotubes were calculated from the differences in glucose concentrations between before and after culture.

### 3.10. Western Blotting

L6 myotubes were lysed in Blue Loading Buffer for 1 min after washing with ice-cold PBS. The lysates were sonicated for 10 s, boiled at 100 °C for 10 min and centrifuged at 15,000 rpm for 5 min. The protein concentrations of the supernatants were determined by a commercial assay kit (RC DC Protein Assay, Bio-Rad laboratories Inc., Hercules, CA, USA). Equal amounts of protein samples (50 µg/lane) were electrophoresed on 10% Mini-PROTEAN TGX precast gels (Bio-Rad laboratories Inc., Hercules, CA, USA) and transferred to nitrocellulose membranes (Bio-Rad laboratories Inc., Hercules, CA, USA). The membranes were washed with Tris buffered saline (TBS) for 15 min and blocked with 3% nonfat dry milk, or 80% BSA in TBS containing 0.05% Tween 20 (TBST), at room temperature for 1 h. The membranes were then washed with TBST and incubated with anti-AMPK or anti-phosphorylated AMPK primary antibodies (1:1000 or 1:2000 in blocking buffer, respectively) at 4 °C overnight. The membranes were then washed with TBST and incubated with HRP-linked anti-rabbit IgG secondary antibodies (1:2000 in blocking buffer) at room temperature for 1 h. After washing with TBST, immunoreactive bands were detected by a chemiluminescent reagent (Clarity Western ECL Substrate, Bio-Rad laboratories Inc., Hercules, CA, USA) and quantified by densitometry analysis using a ChemiDoc XRS Plus system (Bio-Rad laboratories Inc., Hercules, CA, USA) and Image Lab software (version 5.2, Bio-Rad laboratories Inc., Hercules, CA, USA).

## 4. Conclusions

Three new malyngamide analogs, 6,8-di-*O*-acetylmalyngamide 2 (**1**), 6-*O*-acetylmalyngamide 2 (**2**), and *N*-demethyl-isomalyngamide I (**3**) were isolated from the marine cyanobacterum *Moorea producens* collected at Okinawa Prefecture, Japan. The gross structures of these compounds were determined on the basis of spectroscopic analysis. The absolute stereostructures of **1** and **2** were established by NOESY experiments, and chemical derivatization and degradation. The relative configuration of the cyclohexanone ring in **3** was determined by a NOESY experiment. These compounds stimulated glucose uptake in cultured L6 myotubes. Compound **1** was found to activate an insulin-independent AMPK pathway, so **1** may have antidiabetic properties.

## Figures and Tables

**Table 1:** Nuclear magnetic resonance (NMR) spectral data for compound **1** and **2** in CDCl_3_.

Position	1			2		
	δ_C_^a^	δ_H_, Mult (*J* in Hz) ^b^	HMBC	δ_C_^a^	δ_H_, Mult (*J* in Hz) ^b^	HMBC
1a	41.6	3.95, ddd (16.5, 5.8, 1.8)	2, 3, 4, 1’	41.7	3.95, ddd (16.3, 5.6, 1.6)	2, 3, 4, 1’
1b		4.23, dd (16.5, 6.7)	2, 3, 4, 1’		4.24, dd (16.3, 6.8)	2, 3, 4, 1’
2	136.3	-	-	136.5	-	-
3	121.6	6.29, d (1.8)	1, 2, 4	121.4	6.27, d (1.6)	1, 2, 4
4	56.1	4.27, s	1, 2, 3, 5, 9, 10	55.1	4.36, s	1, 2, 3, 5, 9, 10
5	201.9	-	-	203.3	-	-
6	72.4	5.41, dd (12.9, 6.7)	5, 7, 6-OAc	72.7	5.60, dd (12.9, 6.8)	5, 7, 6-OAc
7a	31.3	2.32, m	5, 6, 8, 9	34.1	2.26, m	5, 6, 9
7b		2.60, ddd (13.3, 12.9, 2.6)	6		2.54, dt (12.9, 2.3)	5, 6
8	74.8	5.11, dd (2.6, 2.6)	4, 6, 9, 10, 8-OAc	73.8	3.90, dd (6.1, 3.0)	4, 6, 9, 10
9	78.9	-	-	80.4	-	-
10	23.5	1.26, s	4, 5, 8, 9	23.9	1.34, s	4, 8, 9
NH	-	6.68, dd (6.7, 5.8)	1, 1’	-	6.84, dd (6.8, 5.6)	1, 1’
1’	173.8	-	-	173.8	-	-
2’	36.2	2.27, m	1’, 3’	36.2	2.27, m	1’, 3’, 4’
3’	28.7	2.28, m	4’	28.7	2.32, m	1’, 2’, 4’, 5’
4’	130.7	5.45, m	3’, 6’	130.7	5.45, m	2’, 3’, 5’
5’	128.3	5.47, m	4’, 7’	128.1	5.47, m	3’, 4’, 7’
6’	36.3	2.18, m	4’, 5’, 7’, 8’	36.3	2.19, m	4’, 5’, 7’, 8’
7’	80.7	3.17, m	5’, 8’, 9’, 15’	80.8	3.17, m	5’, 8’, 9’, 15’
8’	33.3	1.42, m	6’, 7’, 9’, 10’	33.4	1.43, m	7’, 9’, 10’
9’	25.5	1.27, m ^e^	-	25.5	1.26, m ^f^	-
10’	29.9	1.27, m ^e^	-	29.9	1.26, m ^f^	-
11’	29.4	1.27, m ^e^	-	29.4	1.26, m ^f^	-
12’	32.0 ^c^	1.27, m ^e^	-	31.9 ^d^	1.26, m ^f^	-
13’	22.8 ^c^	1.27, m ^e^	-	22.8 ^d^	1.26, m ^f^	-
14’	14.2	0.87, t (6.7)	12’, 13’	14.2	0.87, t (6.8)	12’, 13’
15’	56.5	3.31, s	7’	56.5	3.31, s	7’
6-OAc	170.3	-	-	170.4	-	-
	20.8	2.16, s	6-OAc	20.9	2.15, s	6-OAc
8-OAc/OH	169.9	-	-	-	3.00, d (3.9)	7, 8, 9
	21.3	2.19, s	8-OAc	-	-	-
9-OH	-	5.69, s	8, 9, 10	-	5.33, s	8, 9, 10

^a^ Recorded at 125 MHz. ^b^ Recorded at 500 MHz. ^c,d^ Assignments may be interchanged. ^e,f^ Overlapped signals.

**Table 2:** NMR spectral data for compound **3** in CDCl_3_.

Position	δ_C_^a^	δ_H_, mult (*J* in Hz) ^b^	HMBC
1	37.3	4.08, d (6.1)	2, 3, 4, 1’
2	135.1	-	-
3	120.9	6.29, s	1, 2, 4
4	61.4	-	-
5	204.2	-	-
6	51.0	2.41, m	5, 7, 8, 10
7	68.7	3.81, m	6, 8, 9, 10
8a	31.7	2.08, ddd (14.9, 8.5, 2.0)	6, 7, 9
8b		2.61, ddd (14.9, 4.7, 2.6)	4, 6, 7
9	62.1	3.26, dd (2.6, 2.0)	4, 7, 8
10	12.4	1.24, d (7.0)	5, 6, 7
NH	-	6.03, t (6.1)	1, 1’
1’	173.0	-	-
2’	36.4	2.21, m	1’, 3’, 4’
3’	28.6	2.30, m	2’, 4’, 5’
4’	130.7	5.45, m	3’, 5’ 6’
5’	128.0	5.47, m	3’, 4’, 6’, 7’
6’	36.4	2.18, m	7’, 8’
7’	80.8	3.16, m	5’, 8’, 9’, 15’
8’	33.5	1.42, m	7’, 9’, 10’
9’	25.5	1.27, m ^d^	-
10’	29.9	1.27, m ^d^	-
11’	29.4	1.27, m ^d^	-
12’	32.0 ^c^	1.27, m ^d^	-
13’	22.8 ^c^	1.27, m ^d^	-
14’	14.2	0.88, t (6.8)	12’, 13’
15’	56.6	3.31, s	7’

^a^ Recorded at 125 MHz. ^b^ Recorded at 500 MHz. ^c^ Assignments may be interchanged. ^d^ Overlapped signals.

## Supplementary Materials

The following are available online at www.mdpi.com/1660-3397/15/12/367/s1, Figures S1–S6: ^1^H NMR, ^13^C NMR, HSQC, HMBC, COSY, and NOESY spectrum of 6,8-di-*O*-acetylmalyngamide 2 (**1**) in CDCl_3_; Figures S7–S12: ^1^H NMR, ^13^C NMR, HSQC, HMBC, COSY, and NOESY spectrum of 6-*O*-acetylmalyngamide 2 (**2**) in CDCl_3_; Figures S13–S18: ^1^H NMR, ^13^C NMR, HSQC, HMBC, COSY, and NOESY spectrum of *N*-demethyl-isomalyngamide I (**3**) in CDCl_3_; Figures S19–S23: ^1^H NMR, ^13^C NMR, HSQC, HMBC, and COSY spectrum of compound **4** in CDCl_3_; Figure S24: ^1^H NMR spectrum of acetylated compound of **2** in CDCl_3_; Figure S25: ^1^H NMR spectrum of *S*-MTPA ester of **2** in CDCl_3_; Figure S26: ^1^H NMR spectrum of *R*-MTPA ester of **2** in CDCl_3_; Figure S27: ^1^H NMR spectrum of lyngbic acid from **2** in CDCl_3_; Figure S28: ^1^H NMR spectrum of lyngbic acid from **3** in CDCl_3_, Table S1: NMR spectral data for compound **4** in CDCl_3_.
