# Supplementary material for: Three New Malyngamides from the Marine Cyanobacterium Moorea producens
Source: Mar Drugs. 2017 Nov 29;15(12):367. doi: 10.3390/md15120367 (PMC5742827; doi:10.3390/md15120367)
Supplement: Supplementary file 1 [file marinedrugs-15-00367-s001.pdf]

Supplementary Materials: Three New Malyngamides from the Marine Cyanobacterium *Moorea producens*

Kosuke Sueyoshi, Aki Yamano, Kaori Ozaki, Shimpei Sumimoto, Arihiro Iwasaki, Kiyotake Suenaga and Toshiaki Teruya*

Table S1. NMR spectral data for compound **4** in $CDCl_3$.

Position	δ_C [a]	δ_H, mult (J in Hz) [b]	HMBC
1a	38.0	4.07, dd (15.2, 5.5)	2, 3, 4, 1′
1b		4.19, dd (15.2, 6.4)	2, 3, 4, 1′
2	135.3		
3	121.0	6.37, s	1, 2, 4
4	62.1		
5	194.2		
6	133.2		
7	138.5	6.34, m	
8a	27.3	2.76, m	7
8b		2.89, m	6, 7
9	61.3	3.52, m	7, 8
10	16.5	1.83, d (1.1)	5, 6, 7
NH		5.98, dd (6.4, 5.5)	1′
1′	172.5		
2′	36.5	2.20, m	1′, 3′, 4′
3′	28.8	2.29, m	1′, 2′, 4′, 5′
4′	130.8	5.45, m	2′, 3′, 5′ 6′
5′	127.9	5.47, m	3′, 4′, 6′
6′	36.4	2.18, m	5′, 7′, 8′
7′	80.8	3.14, m	5′, 6′, 8′, 9′, 15′
8′	33.5	1.42, m	6′, 7′, 9′, 10′
9′	25.5	1.27, m [d]	
10′	29.9	1.27, m [d]	
11′	29.5	1.27, m [d]	
12′	32.0 [c]	1.27, m [d]	
13′	22.9 [c]	1.27, m [d]	
14′	14.3	0.88, t (6.9)	12′, 13′
15′	56.7	3.32, s	7′

[a] Recorded at 125 MHz. [b] Recorded at 500 MHz. [c] Assignments may be interchanged. [d] Overlapped signals.

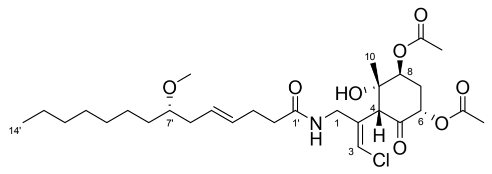

1

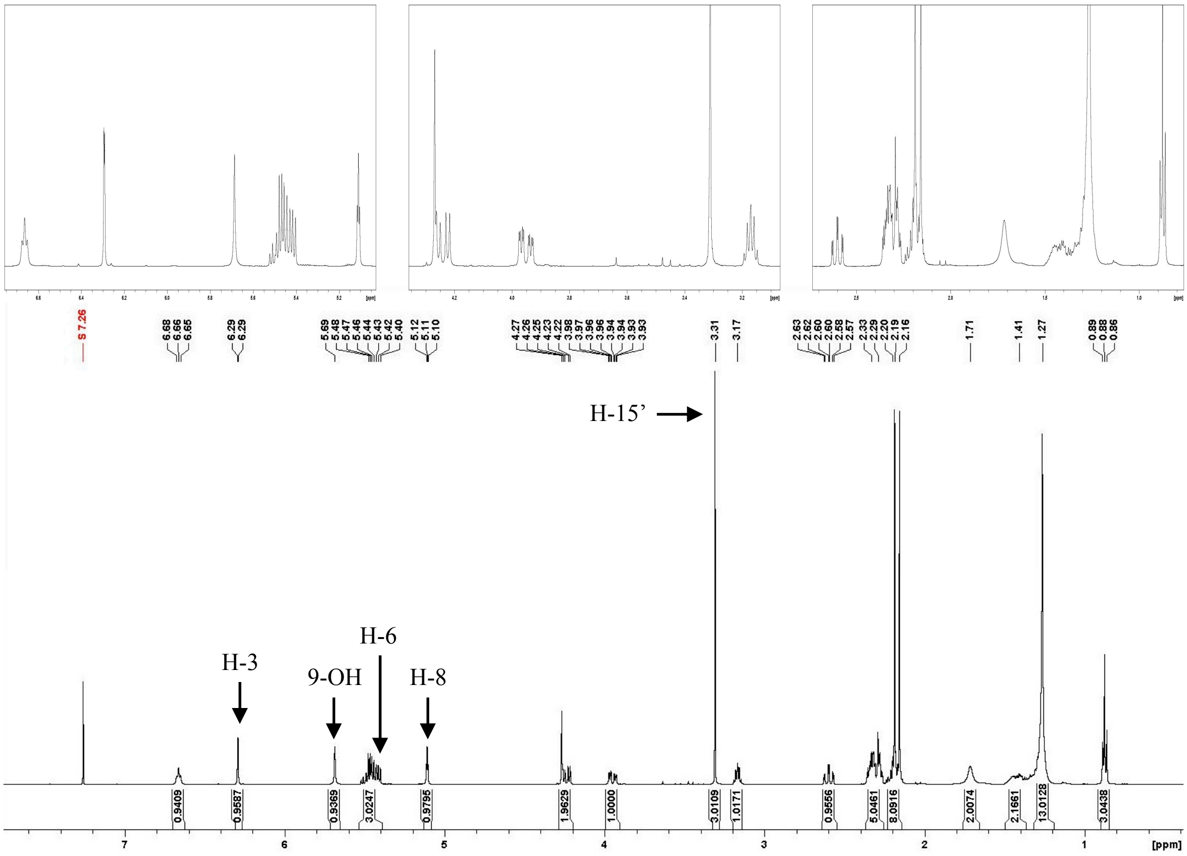

Figure S1. ^{1}H NMR spectrum of 6,8-di-*O*-acetyl-malyngamide 2 (**1**) in $CDCl_3$.

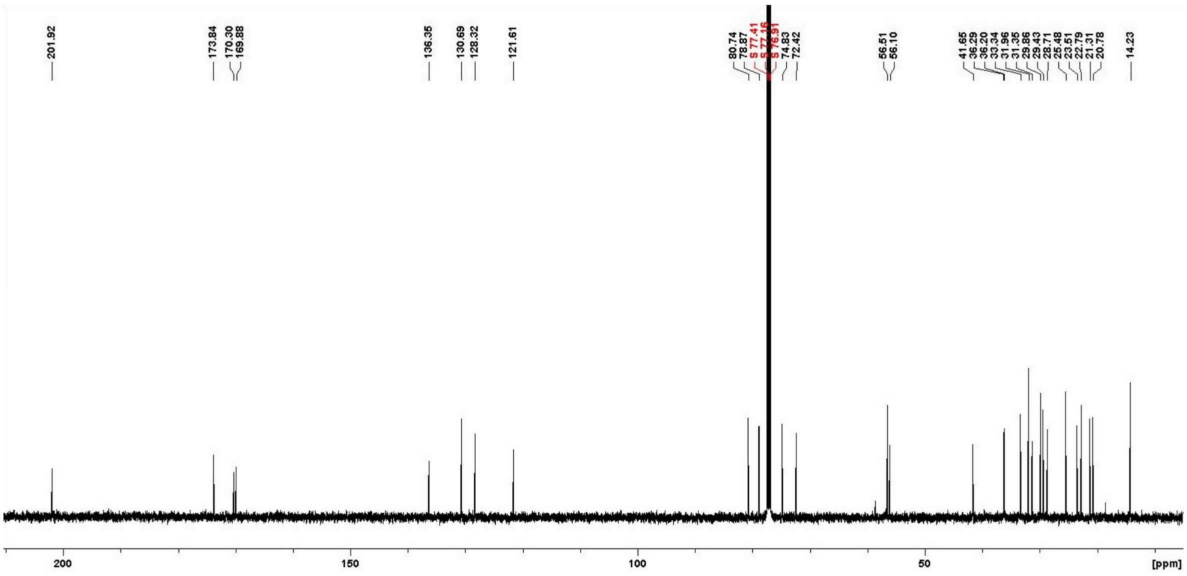

Figure S2. ^{13}C NMR spectrum of 6,8-di-*O*-acetyl-malyngamide 2 (**1**) in $CDCl_3$.

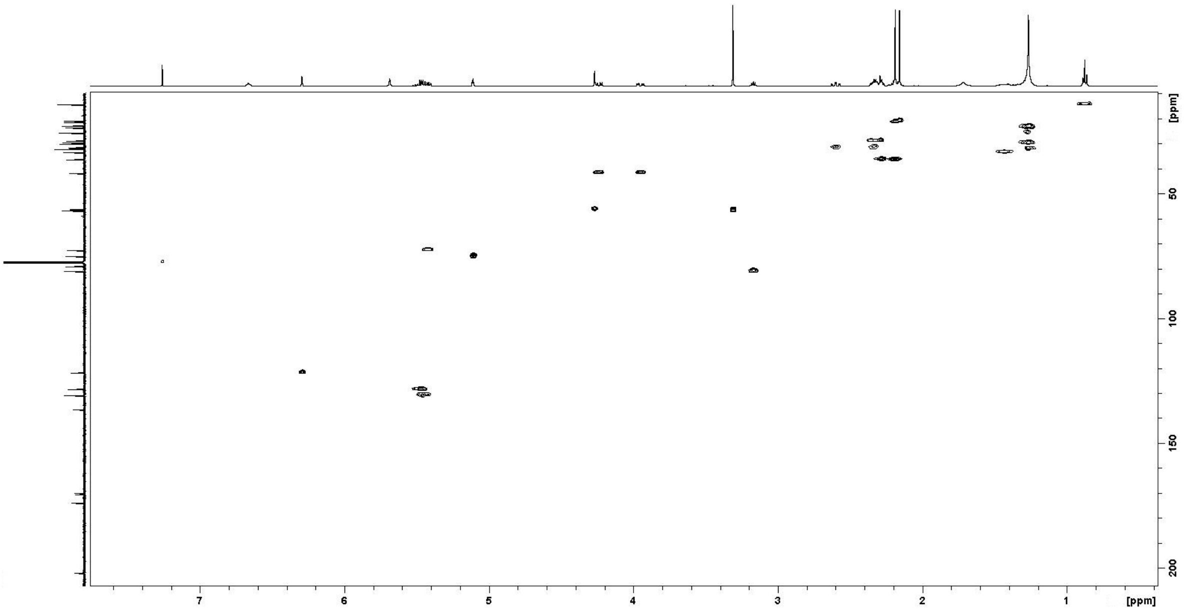

Figure S3. HSQC spectrum of 6,8-di-*O*-acetyl-malyngamide 2 (**1**) in $CDCl_3$.

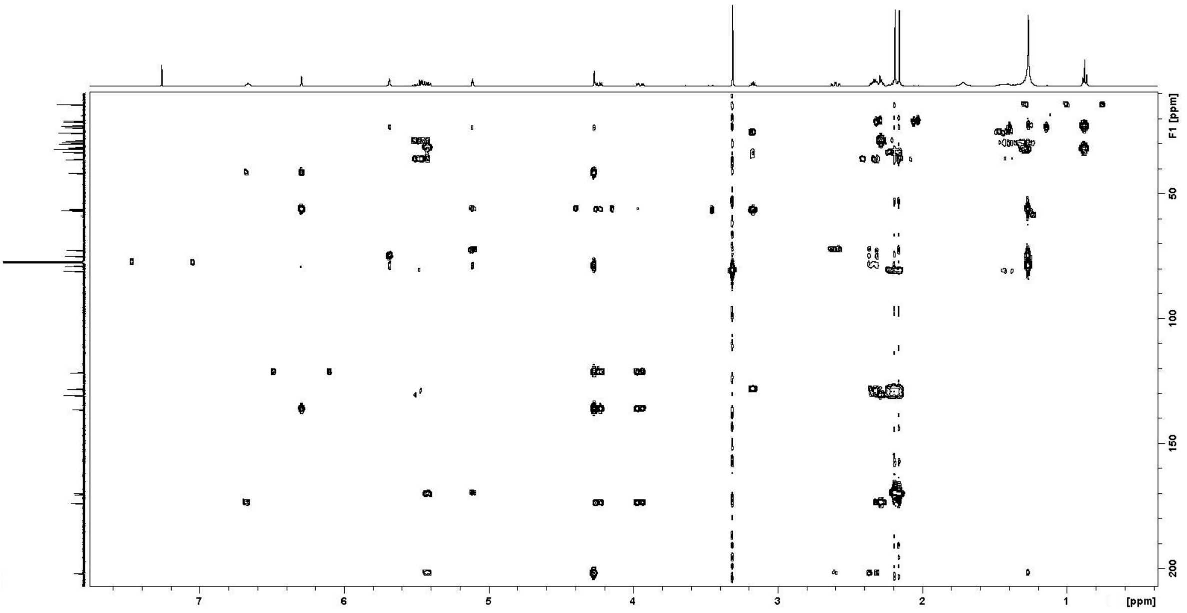

Figure S4. HMBC spectrum of 6,8-di-*O*-acetyl-malyngamide 2 (**1**) in $CDCl_3$.

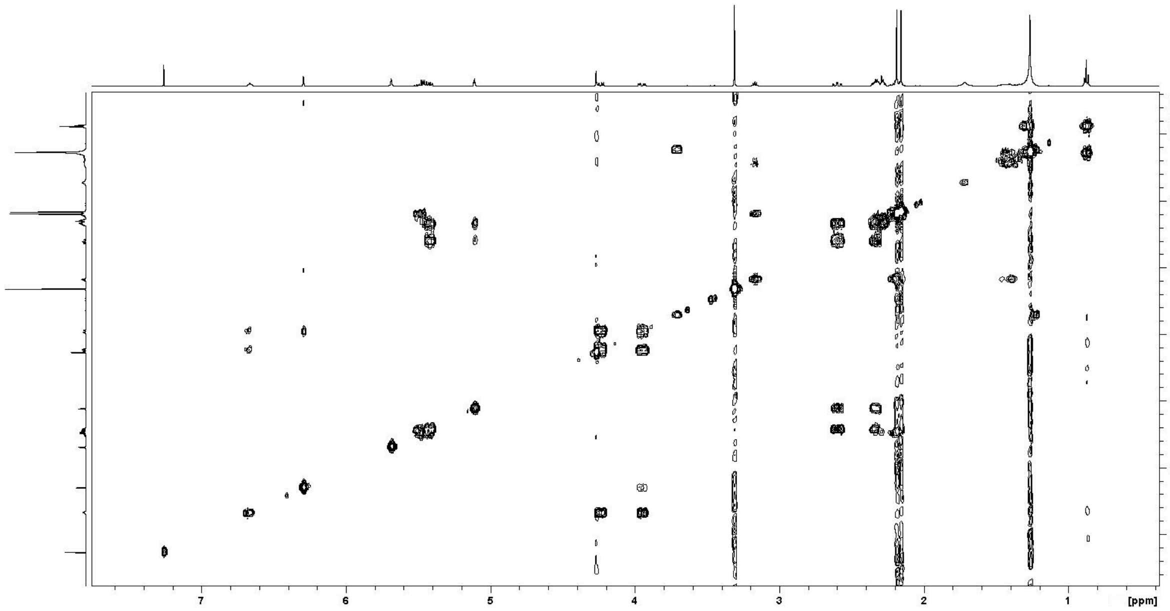

Figure S5. COSY spectrum of 6,8-di-*O*-acetyl-malyngamide 2 (**1**) in $CDCl_3$.

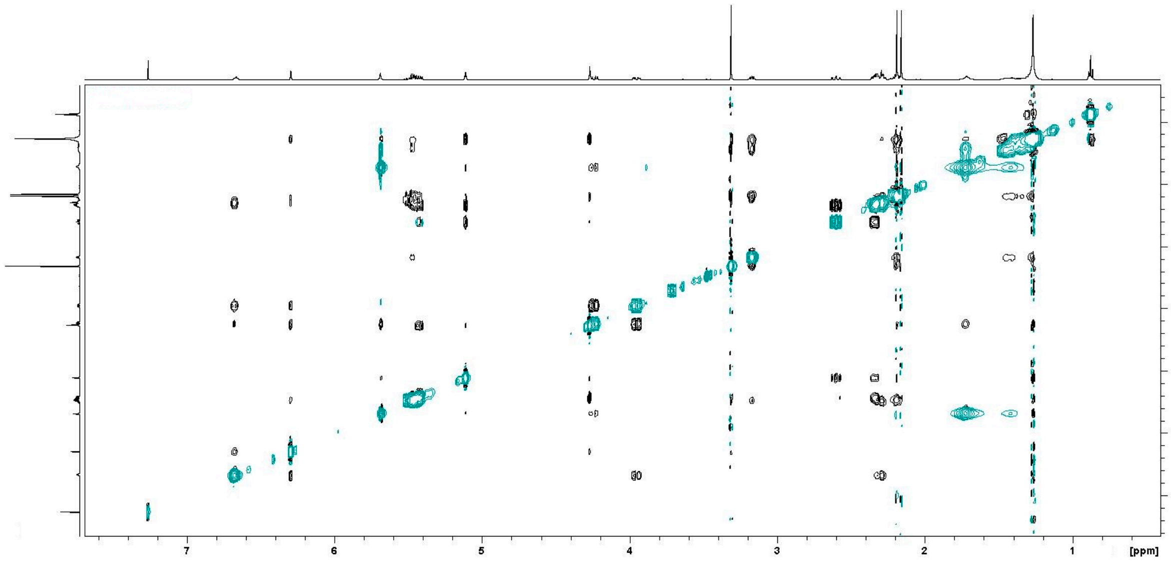

Figure S6. NOESY spectrum of 6,8-di-*O*-acetyl-malyngamide 2 (**1**) in $CDCl_3$.

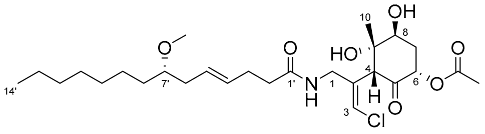

2

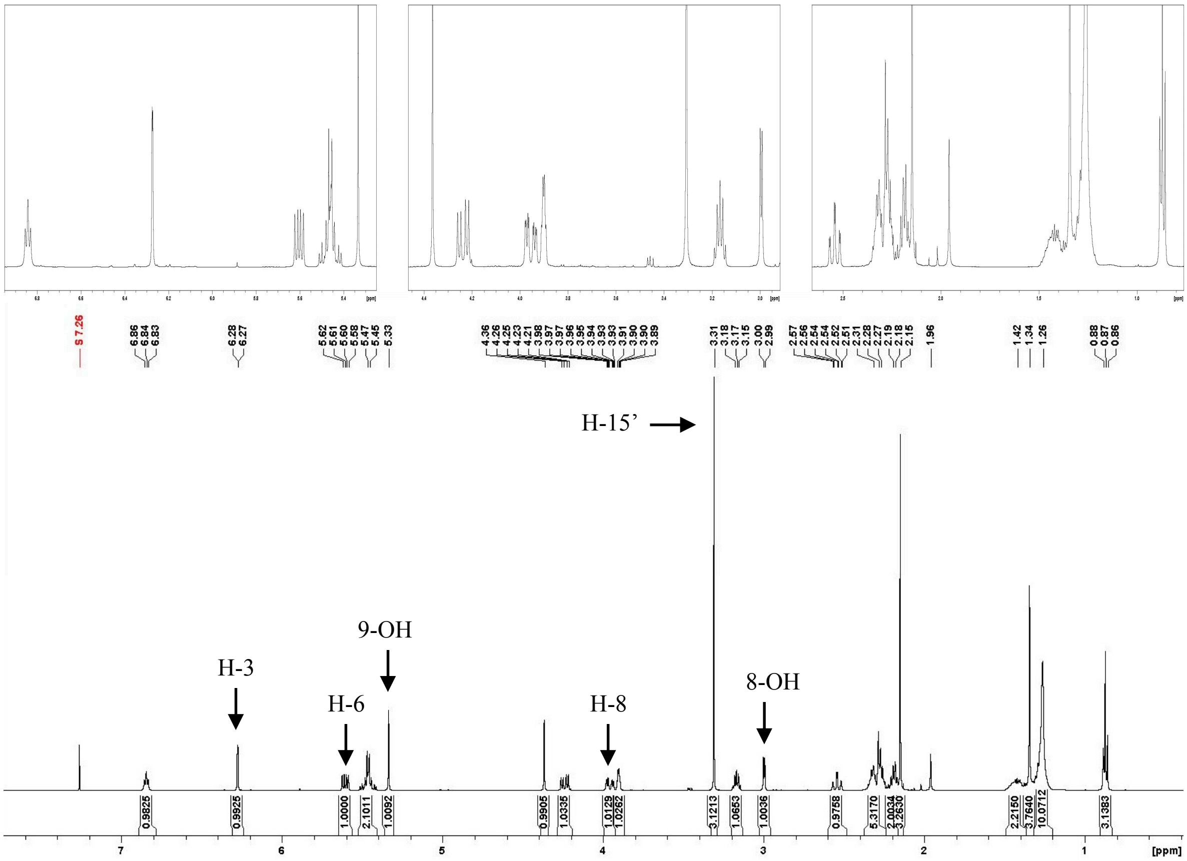

Figure S7. ^{1}H NMR spectrum of 6-*O*-acetyl-malyngamide 2 (**2**) in $CDCl_3$.

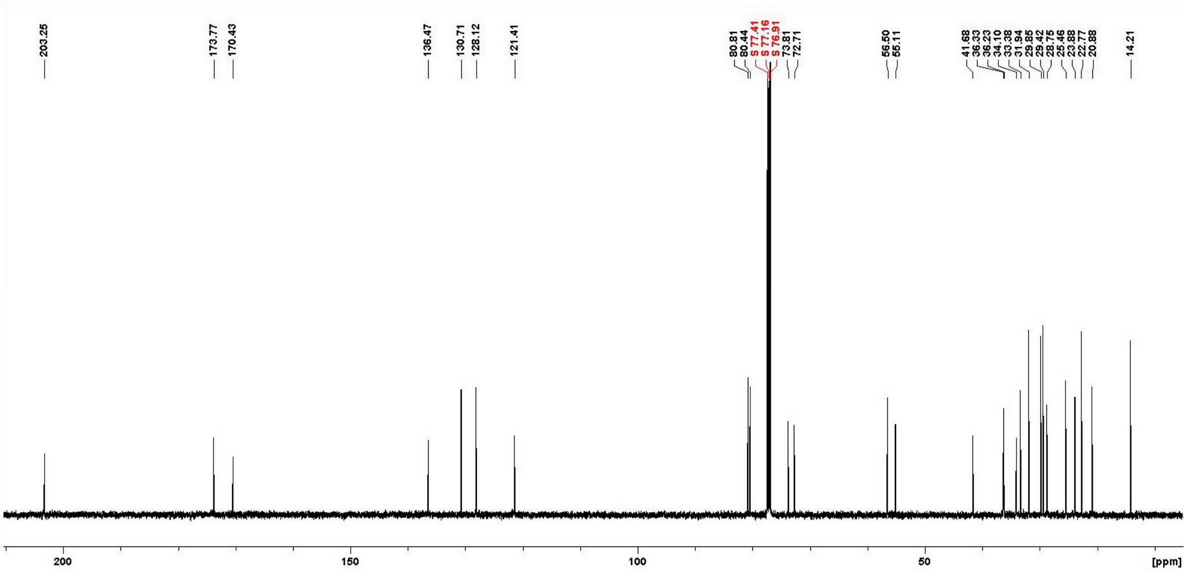

Figure S8. ^{13}C NMR spectrum of 6-*O*-acetyl-malyngamide 2 (**2**) in $CDCl_3$.

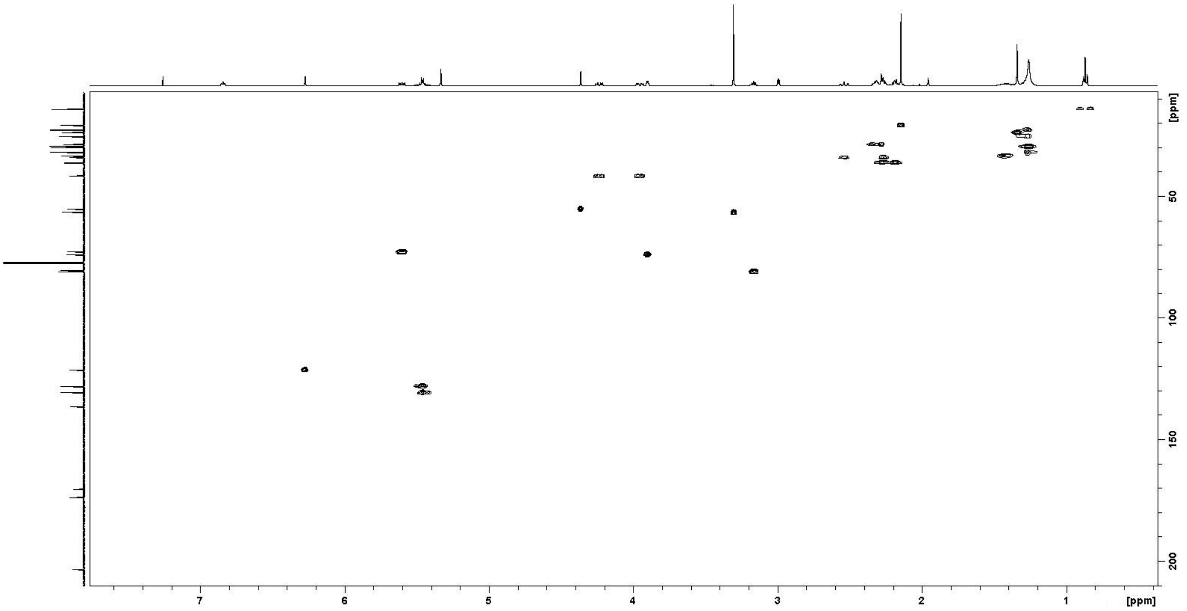

Figure S9. HSQC spectrum of 6-*O*-acetyl-malyngamide 2 (**2**) in $CDCl_3$.

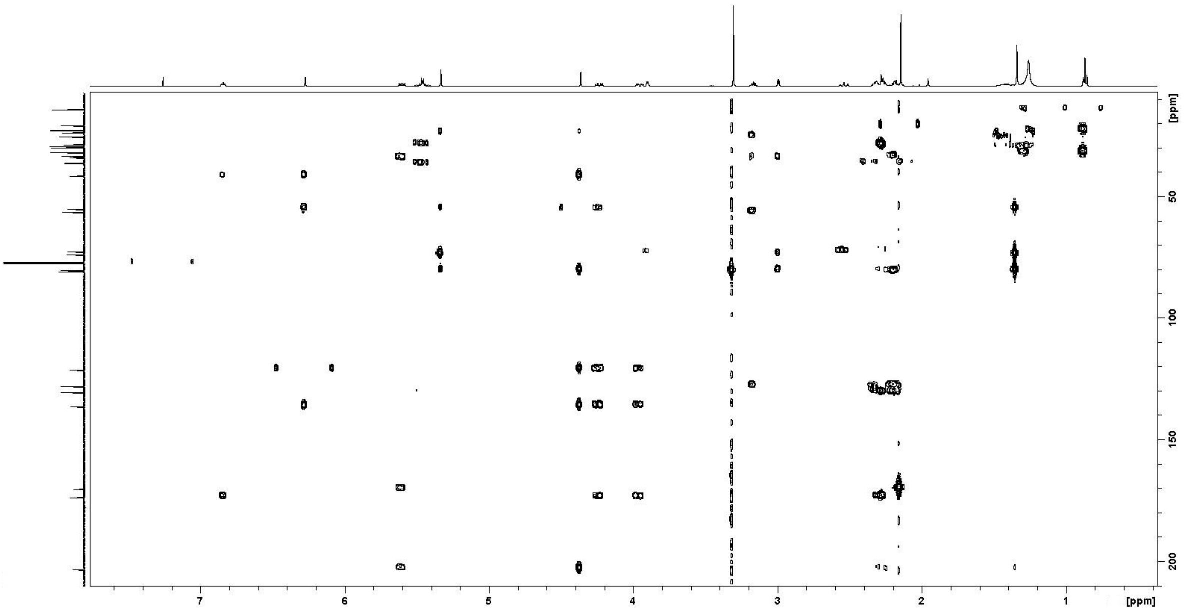

Figure S10. HMBC spectrum of 6-*O*-acetyl-malyngamide 2 (**2**) in $CDCl_3$.

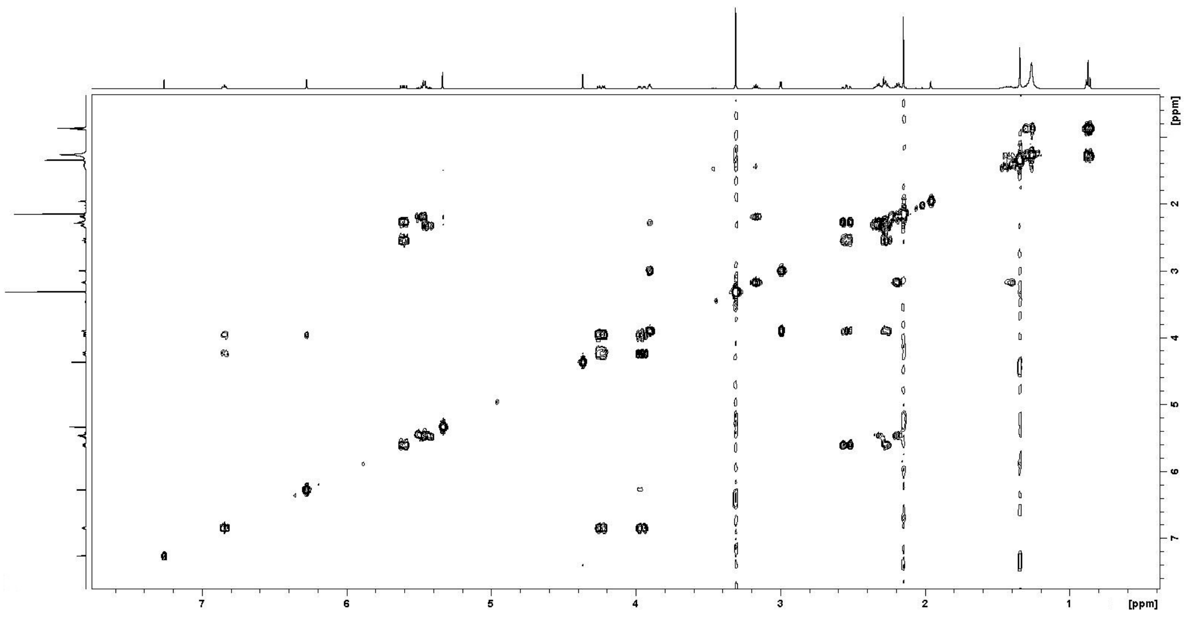

Figure S11. COSY spectrum of 6-*O*-acetyl-malyngamide 2 (**2**) in $CDCl_3$.

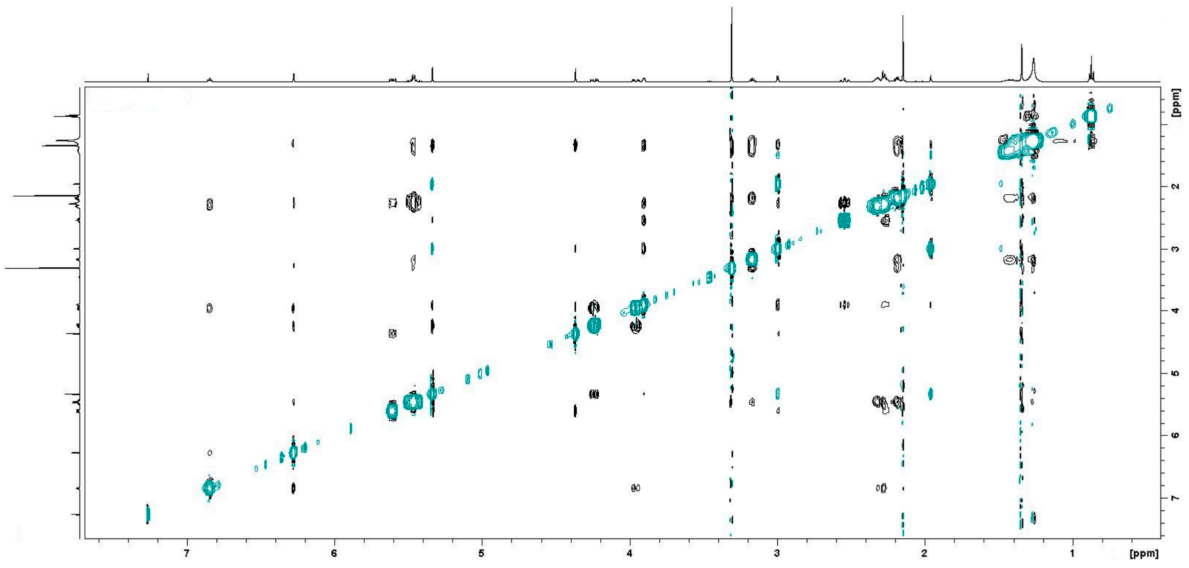

Figure S12. NOESY spectrum of 6-*O*-acetyl-malyngamide 2 (**2**) in $CDCl_3$.

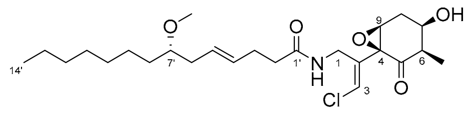

3

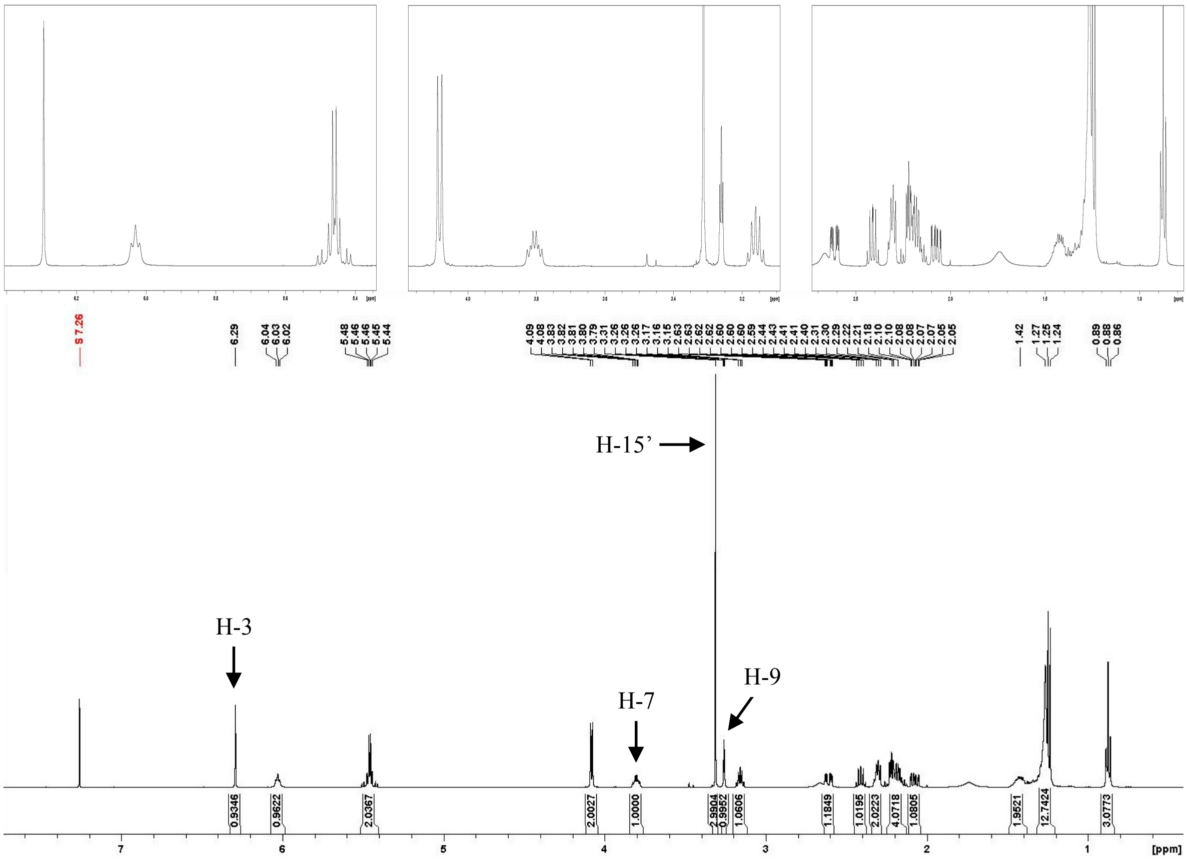

Figure S13. ^{1}H NMR spectrum of *N*-demethyl-isomalyngamide I (**3**) in $CDCl_3$.

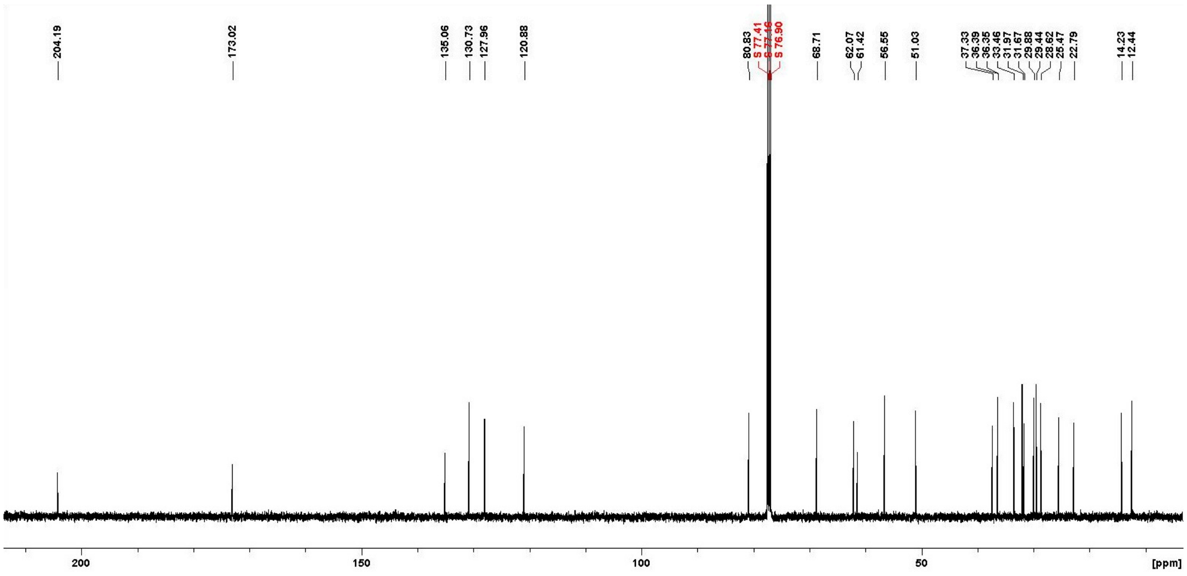

Figure S14. ^{13}C NMR spectrum of *N*-demethyl-isomalyngamide I (**3**) in $CDCl_3$.

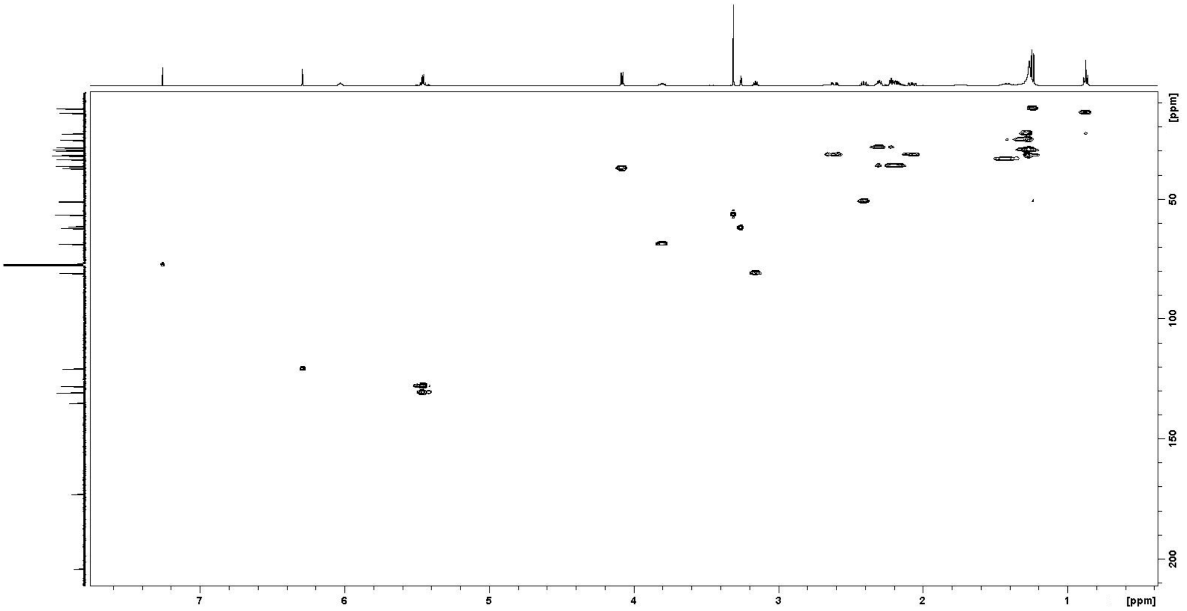

Figure S15. HSQC spectrum of *N*-demethyl-isomalyngamide I (**3**) in $CDCl_3$.

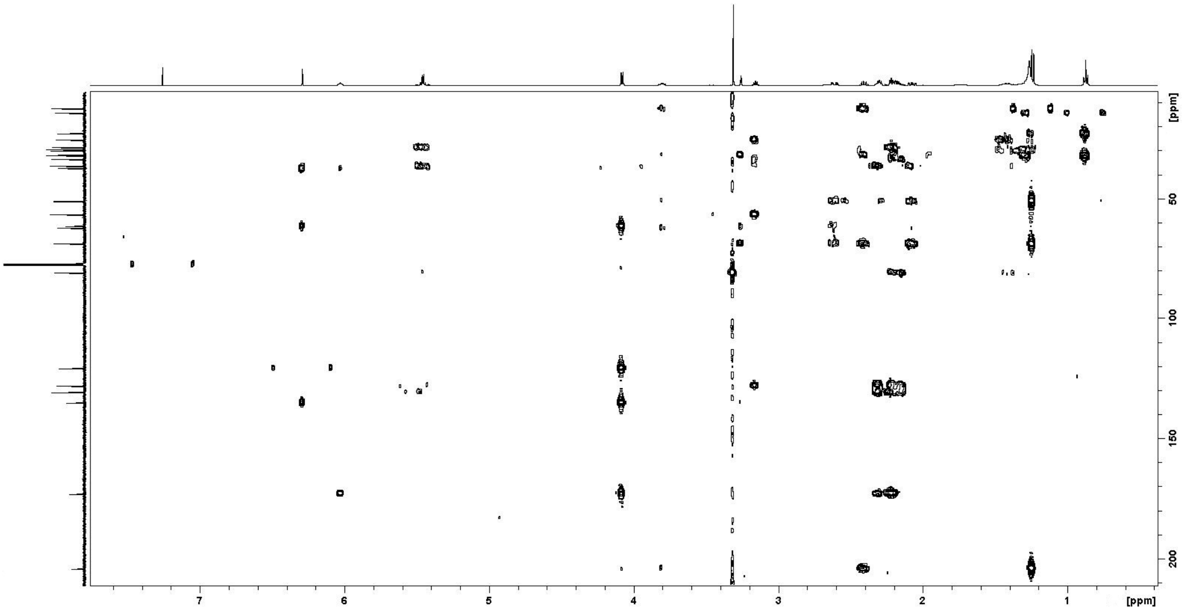

Figure S16. HMBC spectrum of *N*-demethyl-isomalyngamide I (**3**) in $CDCl_3$.

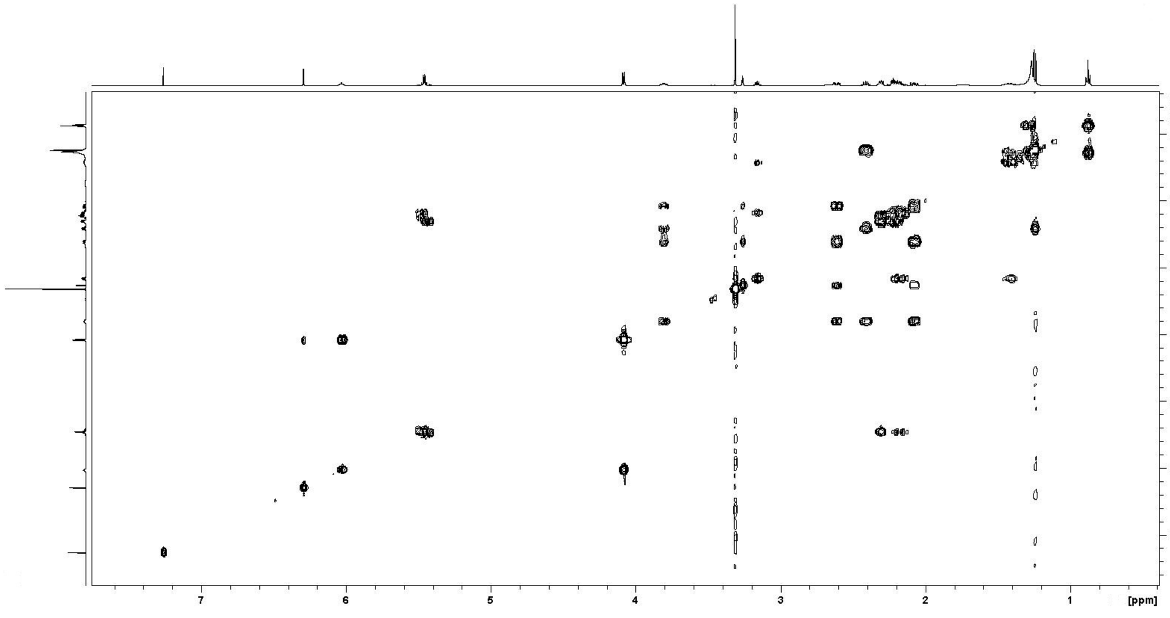

Figure S17. COSY spectrum of *N*-demethyl-isomalyngamide I (**3**) in $CDCl_3$.

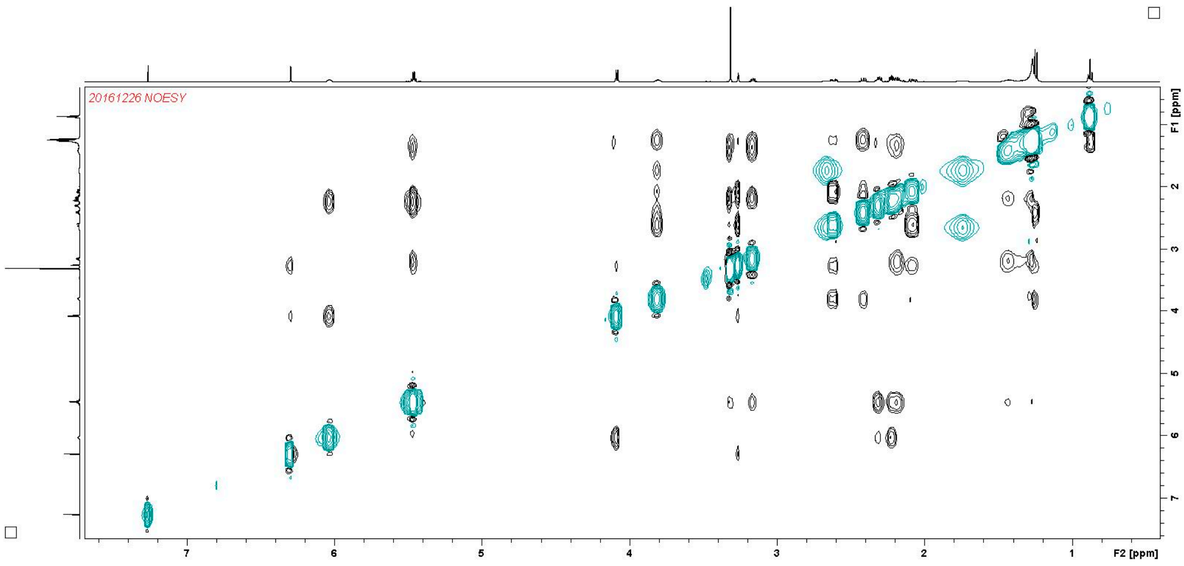

Figure S18. NOESY spectrum of *N*-demethyl-isomalyngamide I (**3**) in $CDCl_3$.

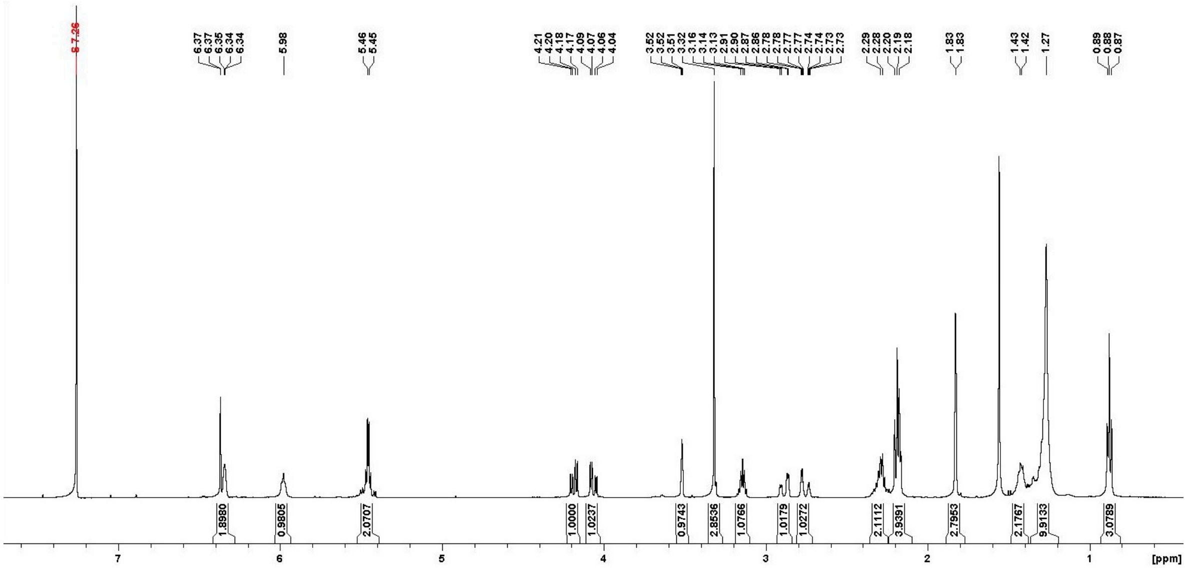

Figure S19. ^{1}H NMR spectrum of compound **4** in $CDCl_3$.

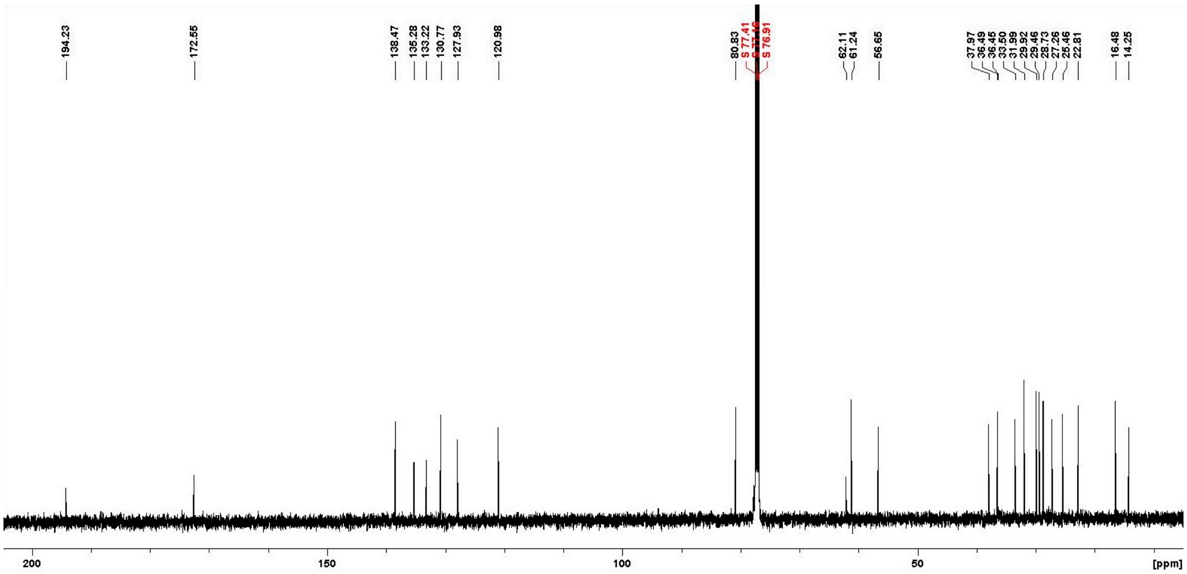

Figure S20. ^{13}C NMR spectrum of compound **4** in $CDCl_3$.

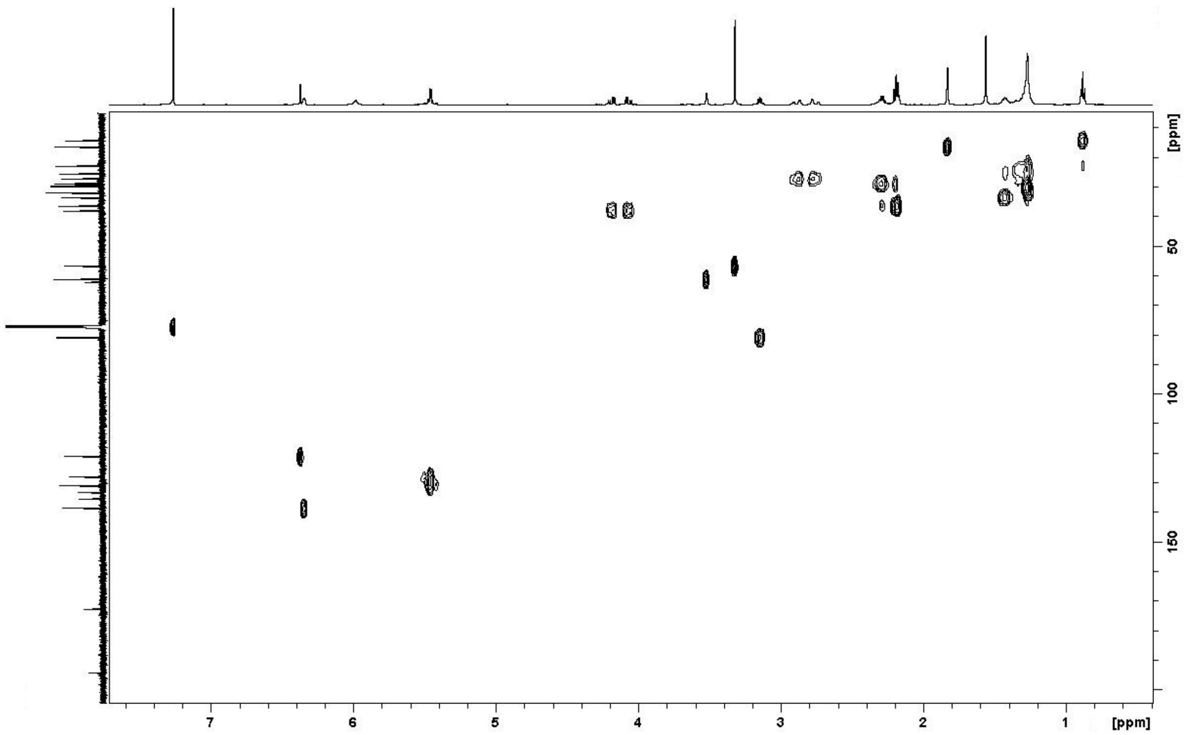

Figure S21. HSQC spectrum of compound **4** in CDCl3.

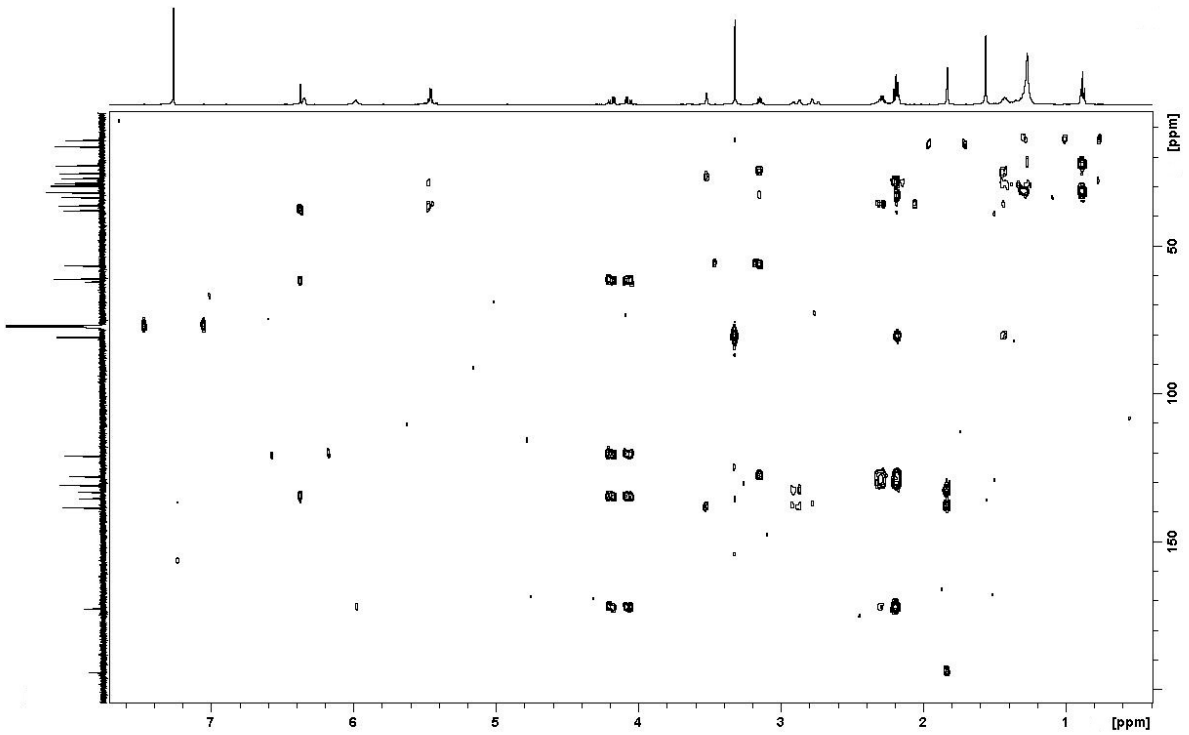

Figure S22. HMBC spectrum of compound **4** in CDCl3.

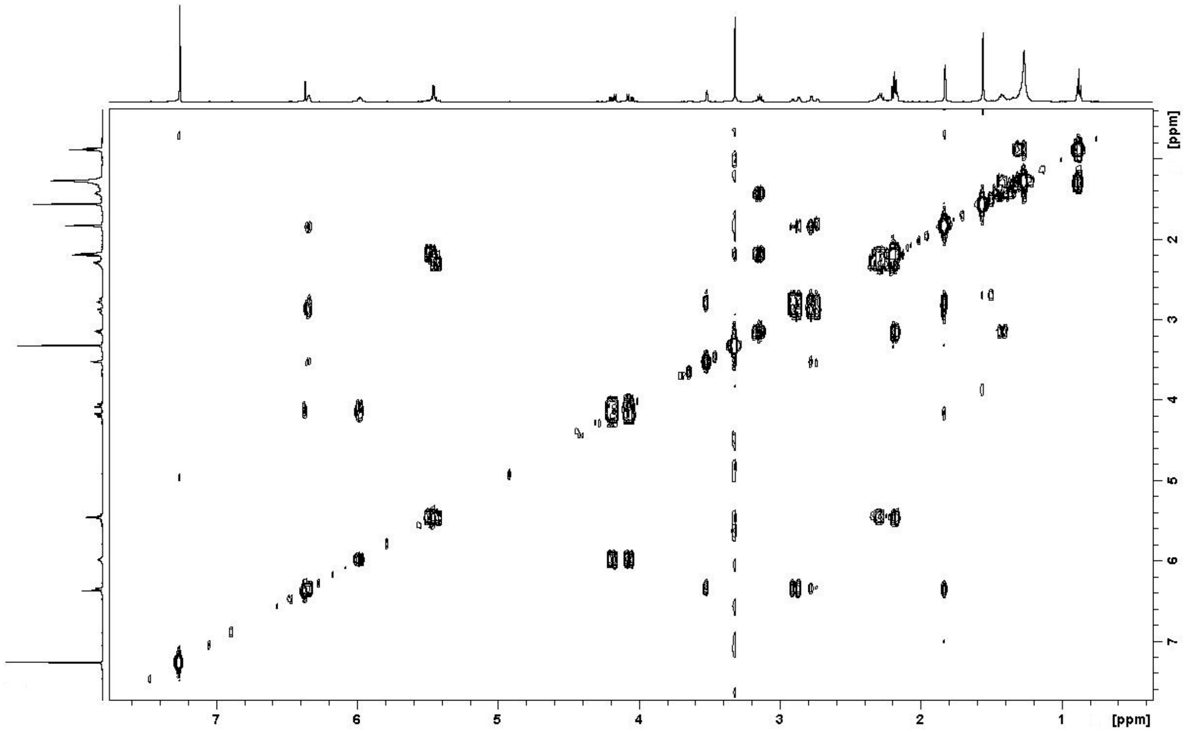

Figure S23. COSY spectrum of compound **4** in CDCl3.

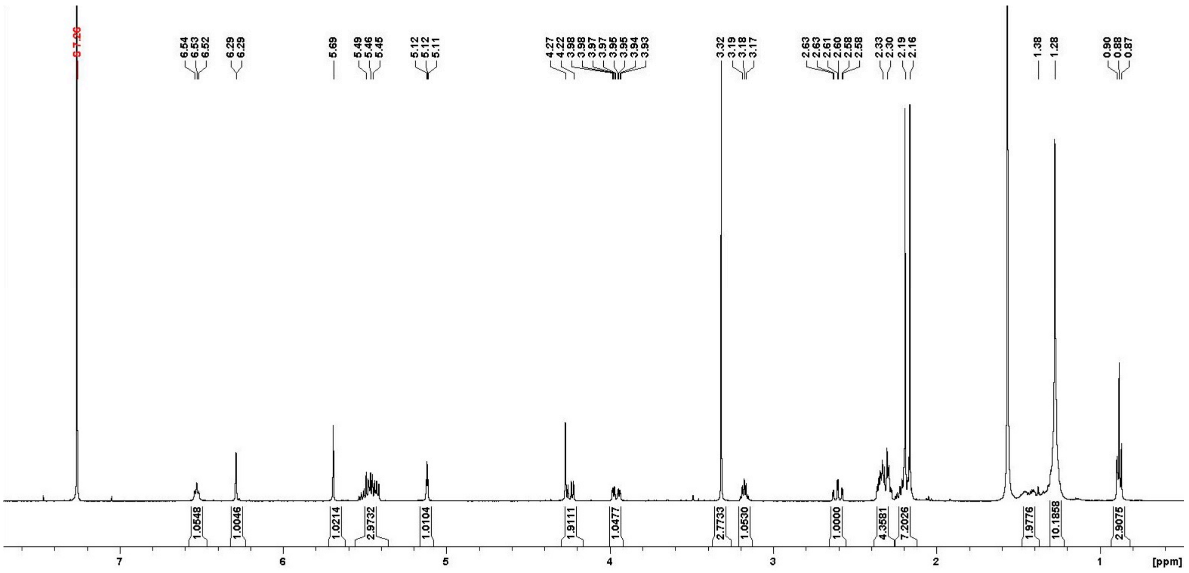

Figure S24. ^{1}H NMR spectrum of acetylated compound of **2** in CDCl3.

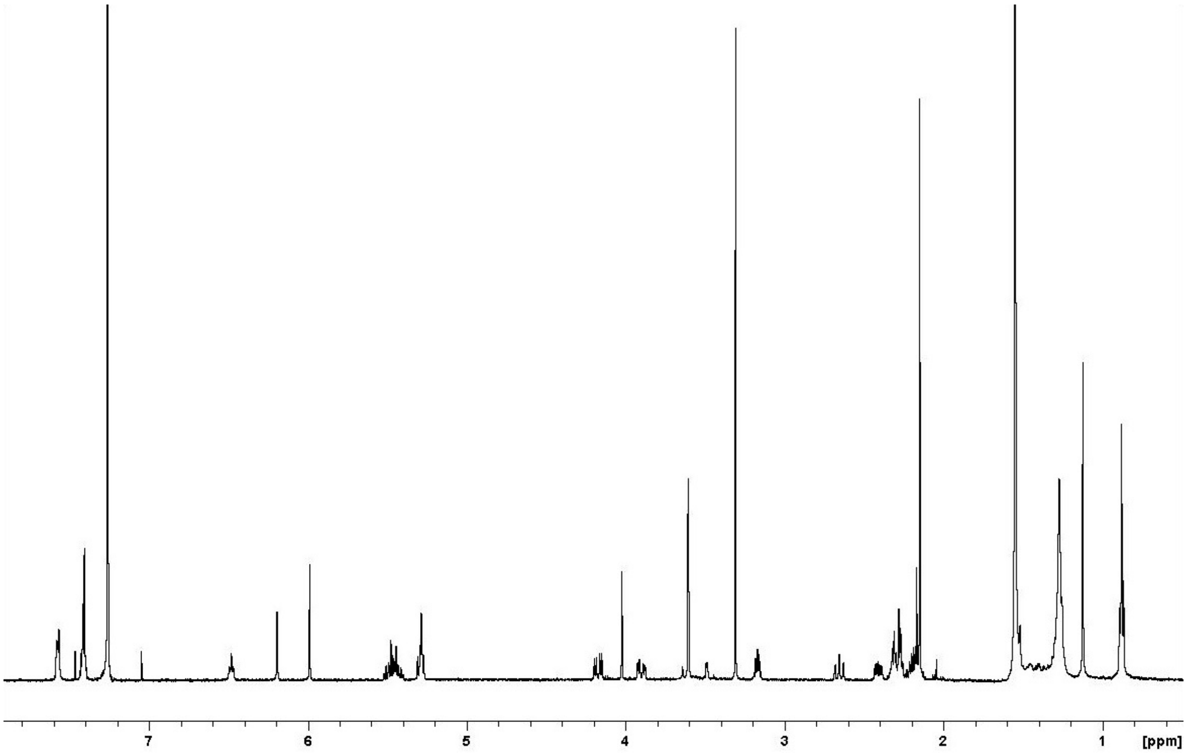

Figure S25. ^{1}H NMR spectrum of *S*-MTPA ester of **2** in $CDCl_3$.

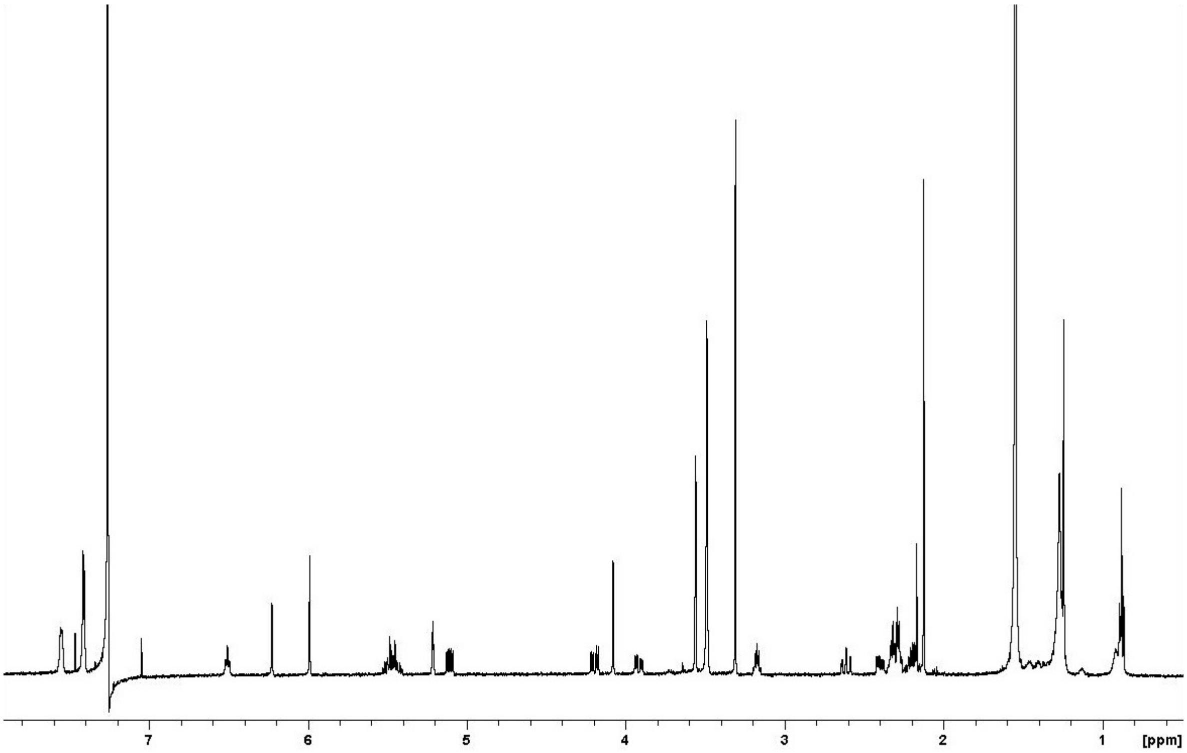

Figure S26. ^{1}H NMR spectrum of *R*-MTPA ester of **2** in $CDCl_3$.

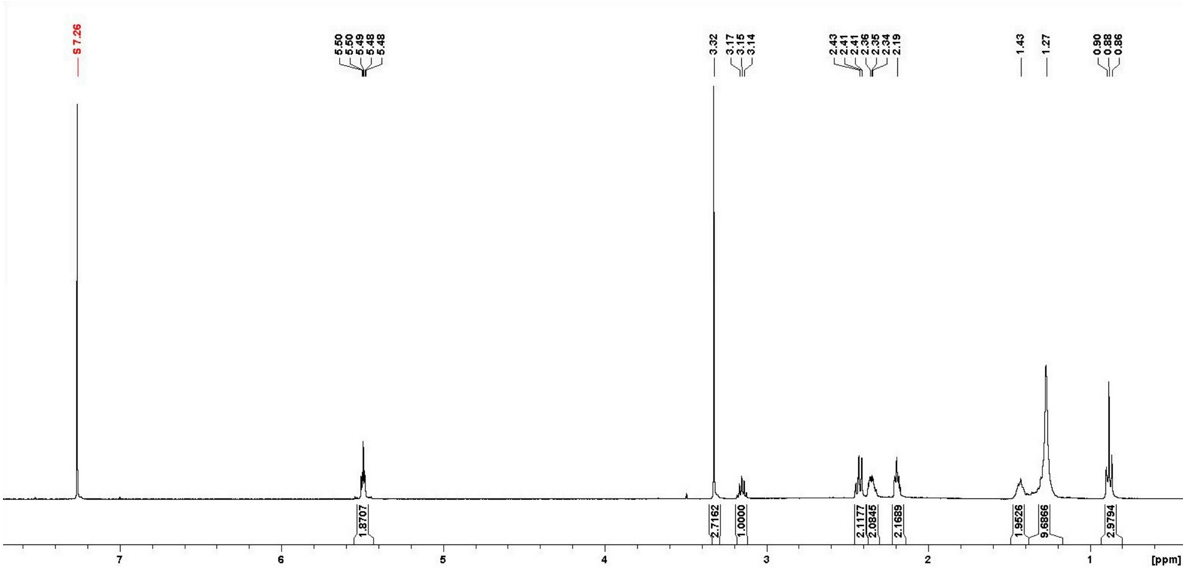

Figure S27. ^{1}H NMR spectrum of lyngbic acid from **2** in $CDCl_3$.

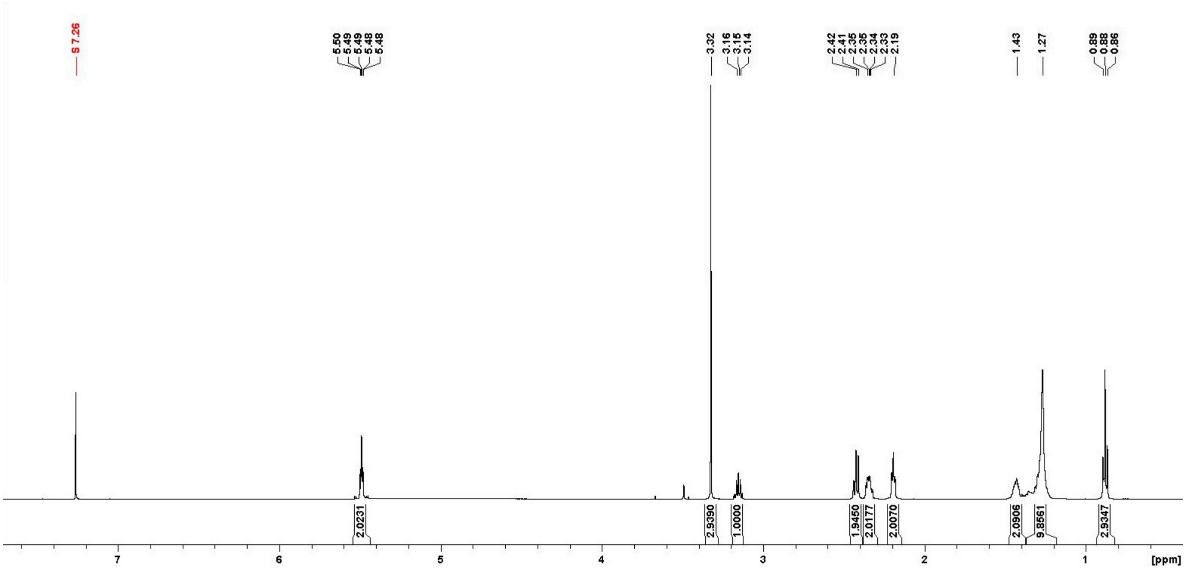

Figure S28. ^{1}H NMR spectrum of lyngbic acid from **3** in $CDCl_3$.
